# Chemical Synthesis and Functional Screening of an 800 000‐Member Library Using a Peptide‐Pairing Strategy

**DOI:** 10.1002/anie.3693802

**Published:** 2026-09-08

**Authors:** Anne Zarda, Xingwang Deng, Edward Will, Thomas Emel, Maylin Romero, Christian Heinis

**Affiliations:** ^1^ Institute of Chemical Sciences and Engineering School of Basic Sciences École Polytechnique Fédérale de Lausanne (EPFL) Lausanne Switzerland

**Keywords:** chemistry, combinatorial chemistry, peptide, peptide library, peptide synthesis

## Abstract

Peptide library generation by chemical synthesis allows using a wide range of non‐canonical amino acids and thus the screening of chemically and structurally highly diverse libraries. However, despite recent improvements in high‐throughput synthesis such as solid‐phase peptide synthesis in 384‐well plates, the size of synthetic peptide libraries is typically limited to a few thousand molecules. Herein we developed a strategy in which *m* short peptides with a thiol functional group are combinatorially paired with *n* short peptides of the same format by a disulfide bridge to generate *m* × *n* different peptide sequences. Using acoustic liquid transfer for rapid reagent transfer and formation of multiple peptide pairs per well in 1536‐well plates, we were able to screen over 800 000 different compounds that led to the identification of nanomolar inhibitors of the coagulation disorder protease thrombin. The concept of pairing synthetic peptide fragments might be applicable to other targets for identifying peptidic ligands below 1 kDa and thereby small enough to potentially deliver starting points for the development of membrane‐permeable drugs for oral application and/or intracellular targeting.

## Graphical abstract

Chemical peptide synthesis allows the generation of structurally highly diverse compounds suited for ligand development, but current methods in parallel peptide synthesis limit the achievable library size to a few thousand. A strategy is presented based on combinatorial pairing of short peptides via disulfide linkage that enables the generation and functional screening of hundreds of thousands of peptides in 1536‐well microwell plates.

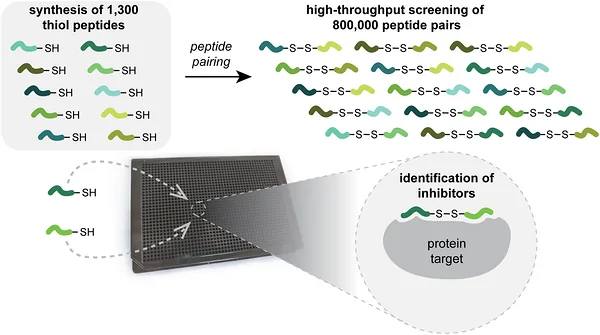

**Abbreviations**
ADE: acoustic droplet ejection
AS‐MS: affinity selection‐mass spectrometry
AUC: area under the curve,
BDT: butanedithiol
DEL: DNA‐encoded chemical library,
DMF: dimethylformamide
Fmoc: fluorenylmethoxycarbonyl,
HTS: high‐throughput screening
K_i_
: inhibitory constant
LC‐MS: liquid chromatography‐mass spectrometry
PPI: protein‐protein interaction
HPLC: high‐performance liquid chromatography
rt: room temperature
SAR: structure activity relationship
SPPS: solid‐phase peptide synthesis
TEA: triethylamine

## 1 Introduction

Combinatorial peptide synthesis allows generating chemically and structurally highly diverse libraries suited for high‐throughput screening (HTS) and ligand development [1, 2]. The modular format of peptides and efficient reactions for peptide bond formation enable automated synthesis of large numbers of peptides in parallel [3, 4]. More than a thousand structurally diverse fluorenylmethoxycarbonyl (Fmoc) amino acids are commercially available for an affordable price [5], and the list of amino acids newly offered is growing rapidly. While ribosomally translated peptides are mostly based on alpha amino acids, chemically synthesized peptides can have highly diverse backbone structures [6, 7, 8], in addition to an enormous structural diversity introduced in the side chains. Chemical library synthesis also offers the flexibility to completely replace peptide bonds by surrogates or to easily introduce functional groups that can be added in ribosomal synthesis only post‐translationally, such as reactive warheads for covalent drug development [9].

Diverse strategies have been developed for generating and screening synthetic peptide libraries [10]. A number of approaches are based on the synthesis of peptides in arrays [11, 12], for example, by SPOT synthesis on membranes [13], by laser printing of reagents to obtain high‐density arrays [14], or by photolithographic methods. Many combinatorial library synthesis methods employ split‐and‐pool synthesis principles, where each bead carries identical copies of one peptide, and binders are either directly identified by screening beads or in affinity selections after release of the peptides [1, 2]. Newer approaches combine split‐and‐pool methods with DNA encoding, allowing identification of binders after affinity selections by DNA sequencing [15, 16]. In some of these approaches, the DNA is additionally exploited for combinatorially pairing synthetic fragments for efficient library generation [17, 18, 19, 20]. Most recently, synthetic peptide libraries were screened in pools by affinity selection‐mass spectrometry (AS‐MS) that does not require the addition of a tag for encoding [21, 22]. Methods based on affinity selection from peptide pools have the advantage that large libraries exceeding a million compounds can be screened, but they also bring challenges such as control of library quality, choice of optimal selection stringency, and correct identification of binders.

Our laboratory has developed methods for the high‐throughput synthesis of peptides in microwell plates, which allows the analysis of individual peptides by liquid chromatography‐mass spectrometry (LC‐MS) and thus easy control of peptide quantity and quality [23, 24]. Importantly, the screening of peptides in microtiter plates enables the testing of compounds using functional assays such as for enzyme inhibition, receptor modulation, or inhibition of protein‐protein interactions (PPI), ensuring that only binders that modulate the target are identified. In addition, functional assays typically offer reliable readouts with usually only small rates of false positive hits. Recently, we established solid‐phase peptide synthesis (SPPS) in 384‐well plates [25] as well as methods for late‐stage chemical diversification at nanomole‐scale, increasing the number of peptides that can be produced and tested in microwell plates to several ten‐thousands [23, 24]. Despite these advances, the size and diversity of the libraries may still be too small for identifying binders to some of the most challenging targets, such as classical antibody targets, of which many could not yet be addressed by orally available small molecule drugs, or to many of the challenging intracellular protein targets, such as PPIs.

Herein, we propose a new strategy for the production and functional screening of much larger libraries of synthetic peptides that is based on combinatorial conjugation of peptide pairs in wells of microwell plates prior to HTS. For example, *m* different peptides are combinatorially linked with another set of *n* peptides to obtain *m* × *n* peptides having a suitable length for ligand development (Figure 1a). We envision using peptides with thiol groups that can be linked by disulfide formation, an efficient and selective reaction that proved suitable for generating dynamic small molecule libraries [26, 27] or combinatorial PROTAC libraries [28]. For generating the vast numbers of combinations, we propose to take advantage of acoustic liquid transfer, a technology in which low nanoliter volumes of reagents can rapidly be transferred from wells of a 384‐ or 1536‐well source plate to wells of choice of a 1536‐well destination plate. With this technology, we can transfer two peptides to wells of 1536‐well plates in volumes as small as 2.5 nanoliters by using only minimal amounts of peptide and without consuming any pipetting tips. The resulting *m* × *n* peptide dimers would contain new combinations of amino acids without the need for their individual synthesis by throughput‐limiting SPPS. We were able to generate libraries of unprecedented size in microwell plates, the largest one having over 800 000 synthetic peptides, from which HTS yielded target‐specific inhibitors of the coagulation target thrombin.

**FIGURE 1:**
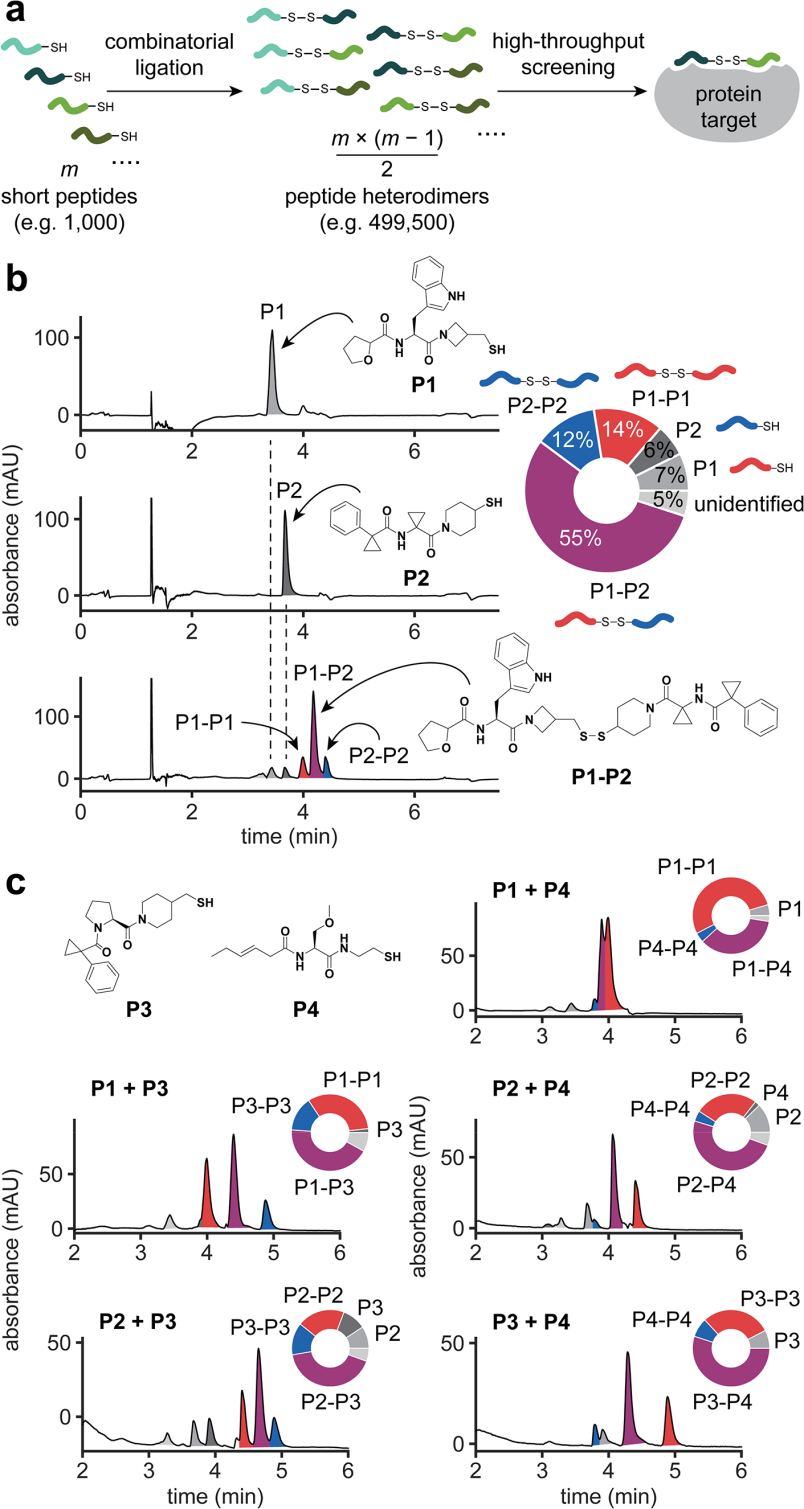
(a) Principle of disulfide peptide pairing strategy to generate large libraries of synthetic peptides. (b) LC‐MS absorbance chromatograms of monomer P1 (top), monomer P2 (middle) and reaction mixture (bottom) recorded at a wavelength of 220 nm. DMSO stocks of the peptides were combined and incubated for 24 h in a 1:1 mixture of DMSO and 120 mM NH_4_HCO_3_ buffer (pH 8) with a final monomer concentration of 100 µM each. The pie diagram shows the percentages of indicated peptide species quantified by the area under the curve (AUC). (c) LC‐MS chromatogram traces of more test reactions with additional peptides P3 and P4 using the same reaction conditions as described for (b). Data is shown for reactions performed with HPLC‐purified peptides. Equivalent data for reactions with crude peptides is shown in Figure S1.

## 2 Results and Discussion

### 2.1 Peptide Pairing by Disulfide Formation

We first tested the covalent pairing of peptides by disulfide bond formation, using solvents and reagents that are compatible with biological assays, the latter being required as we planned to screen the peptide pairs as crude products without purification (Figure 1a). As model peptides, we used high‐performance liquid chromatography (HPLC)‐purified peptides P1 and P2 that were both composed of three building blocks, being an N‐terminal carboxylic acid, an amino acid in the middle, and a C‐terminal aminothiol group (Figure 1b). In a 1:1 solvent mixture of DMSO and 120 mM ammonium bicarbonate buffer (pH 8), the peptides (100 µM each) dimerized nearly quantitatively after incubation for 24 h at room temperature (rt), as assessed by LC‐MS (Figure 1b and Table S1). Quantification based on absorbance showed the product heterodimer P1‐P2 (55%, purple) and the two homodimers P1‐P1 (14%, red) and P2‐P2 (12%, blue), and only small quantities of monomers that remained left (13%, grey) (Figure 1b). The experimental distribution of dimeric products closely matched the expected statistical distribution of 2:1:1 of heterodimer P1‐P2 to homodimer P1‐P1 and homodimer P2‐P2. The heterodimer is present at double concentration because, for a group of equimolar monomers of any size, there are two pairings that yield any heterodimer (P_X_ with P_Y_ and P_Y_ with P_X_), while there is only one pairing that yields any homodimer (P_X_ with P_X_). We performed the reaction using the two additional thiol peptides P3 and P4 and forming all possible combinations, which showed a similar outcome and consistent formation of around 50% heterodimer product, suggesting that the reaction conditions are suitable for peptide‐pair library generation (Figure 1c). We also tested the peptide dimerization with thiol peptides that were not HPLC‐purified after SPPS (84%–96% purity), as hundreds of such crude thiol‐functionalized peptides can easily be produced by parallel peptide synthesis [29], which gave similar results with the desired heterodimers representing 36%–52% of the product (Figure S1).

### 2.2 Solid‐phase Synthesis of 1536 Thiol‐Functionalized Peptides

To generate large combinatorial libraries of disulfide‐paired peptides, we required many short linear peptides, each functionalized with one thiol group. Such peptides can easily be produced using a SPPS strategy recently introduced where peptides are synthesized on a disulfide linker solid support and released by disulfide reduction [29]. In this method, protecting groups are removed and washed away prior to release of the peptide from the solid phase by a reducing agent, which yields crude peptides with a purity of typically > 80%. The only reagents that remain with the released peptides are the reducing agent butanedithiol (BDT), the base triethylamine (TEA), and the solvent dimethylformamide (DMF), which can all be removed under reduced pressure by vacuum centrifugation. Hence, crude peptides do not require precipitation or chromatographic purification, allowing production of hundreds of sequences in 96‐ or 384‐well plates with moderate effort.

We synthesized peptides in four 384‐well reactor plates, yielding a total of 1536 thiol peptide monomers. Each peptide consists of five building blocks, an N‐terminal carboxylic acid, three amino acids in the middle, and an aminothiol group at the C‐terminus (Figure 2a). The sequences were randomly constructed, chosen from a pool of building blocks, including 59 carboxylic acids, 166 Fmoc amino acids (59 in each position), and four aminothiols (Figure 2b,c, Tables S2 and S3). Peptide pairs of this format have an average molecular mass of 1300 Da, which we considered sufficiently large to form many contacts with targets and sufficiently small to potentially convert into a membrane‐permeable compound (Figure S2). We synthesized the peptides on a 1‐µmol scale using polystyrene resin to which we conjugated the first building block (the aminothiol) via a disulfide linker, as recently described [30]. The Fmoc amino acids and terminal carboxylic acids were coupled by automated synthesis, and the peptides were released by reducing the disulfide bridge. A library quality analysis based on 70 randomly selected peptides showed that in over half of all samples (54%), the largest peak in the LC‐MS chromatogram corresponded to the desired peptide, and that in another 15%, the desired peptide could be detected but as a minor peak (Figures S3 and S4, Table S4). In many wells that did not contain the desired peptide, mass spectrometry revealed truncation products lacking one or more building blocks. Because each well contained one and, in many cases, multiple identifiable peptide products, we estimate that the total number of identifiable monomers, and thus the effective diversity of the monomer library, clearly exceeds 1536. To allow mixing of the peptide pairs at equimolar amounts, we determined the concentrations of all peptides using Ellman's assay and prepared 1 mM monomer stocks in a 1536‐well plate from which they could be easily transferred by acoustic liquid transfer.

**FIGURE 2:**
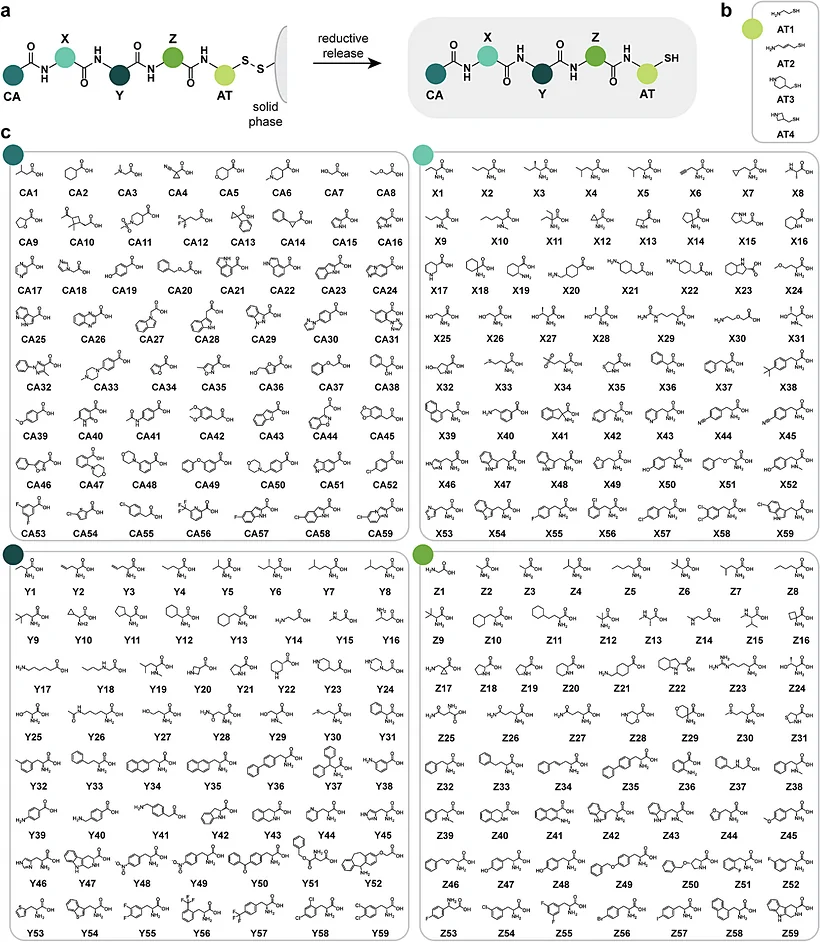
Synthesis of thiol‐functionalized short peptide monomers. (a) Format of peptides and synthesis on disulfide‐linker solid support. (b) Aminothiols used at the C‐terminus of the peptides. (c) Chemical building blocks used for SPPS of the 1536 thiol peptides.

### 2.3 Synthesis of a 19 200‐Member Peptide Pair Library

Combinatorially pairing all the 1536 synthesized peptide monomers with each other, as schematically shown in Figure 3, would yield in total around 1.2 million unique peptide dimers (1536 × 1535/2 heterodimers, plus 1536 homodimers). In this calculation, the number of combinations is divided by two in order to consider only the first occurrence of each pairing (e.g., M1‐M2 and M2‐M1 are identical molecules). If one heterodimer is made per well (two monomers per well), around 800 1536‐well screening plates would be required to synthesize all possible dimers. Instead, we performed an initial proof‐of‐concept screen in which only a small fraction of the possible combinations was made, chosen as follows to create Library 1: peptide monomers M1–M1536 were split into 64 groups such that each contained 24 thiol peptide monomers. For each group, all unique 276 pair‐wise heterodimer combinations were synthesized in separate wells, yielding 19 200 unique dimers for screening, or 1.6% of the theoretically possible 1.2 million. This resulted in triangular heatmaps that align in the full “dimer space” on the diagonal, as depicted in Figure 3, with, for example, triangle 1 consisting of M1–M24, triangle 2 of M25–M48, and so on.

**FIGURE 3:**
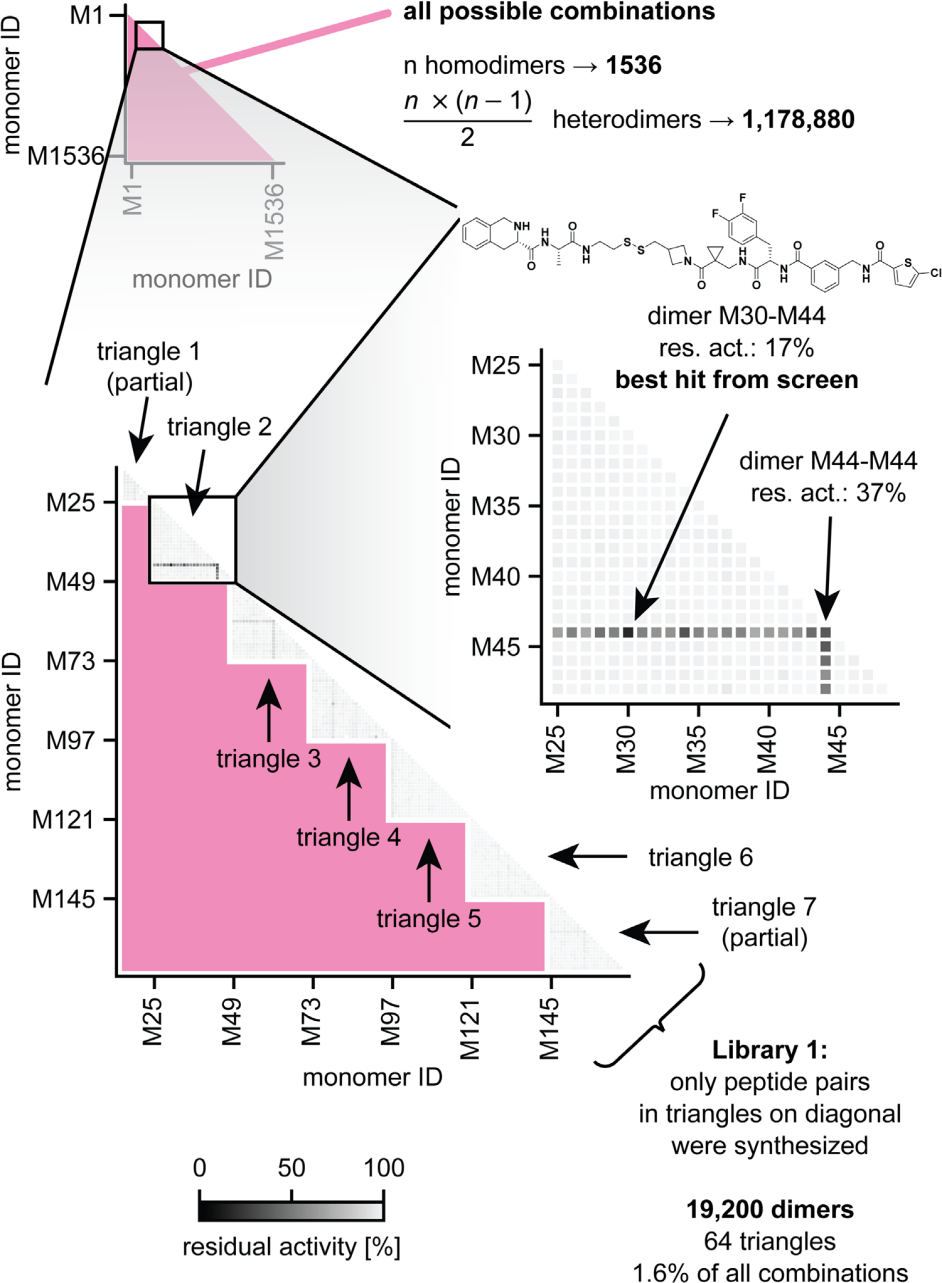
Design of peptide dimer Library 1. Monomers M1–M1536 were organized in 64 groups, each containing 24 monomers. All possible combinations in each group were synthesized and screened against thrombin. Fifteen pmol of each monomer were combined via ADE. Thrombin and assay reagents were added directly to wells of the peptide pair synthesis plate to reach a peptide heterodimer concentration of around 1 µM. A region showing 7 of the 64 groups (triangles 1–7) is shown enlarged. Of this region, one group (triangle 2) is further enlarged to see the activities in individual wells. Triangles with the best hits are shown in Figure 4 and all triangles are shown in the Supporting Information.

We combined the peptide pairs in separate wells of 1,536‐well plates using acoustic droplet ejection (ADE; see workflow in Figure S5). We transferred 15 pmol of each monomer (15 nL each), added ammonium bicarbonate buffer and DMSO (1:1), and left them reacting for 24 h at rt (150 nL final volume; 100 µM final peptide concentration; Table S1). We screened the 19 200 peptide pairs against the target thrombin, a trypsin‐like serine protease implicated in various thrombotic disorders [31]. Thrombin has a well‐defined substrate‐binding pocket against which numerous peptide‐based inhibitors have already been identified, making it a relatively straightforward proof‐of‐concept target. Another advantage of thrombin is the availability of a robust functional screening assay based on a fluorogenic peptide substrate. For assessing thrombin inhibition, we dispensed thrombin and subsequently a fluorogenic thrombin substrate directly to the 1536‐well plates containing the peptide pairs. The target and assay reagents were added at a volume of 7.5 µL that diluted each peptide monomer to a concentration of 2 µM, resulting in a heterodimer concentration of around 1 µM. The screen showed many wells with substantially reduced thrombin activity (> 50% inhibition). In the thrombin inhibition heatmaps shown in Figures 3 and 4, many of the active peptide combinations are arranged in horizontal and vertical lines that indicated dimers that shared one of the two monomers, which we expected was dominating the binding interaction (e.g., M44 shown in the zoomed‐in region in Figure 3).

**FIGURE 4:**
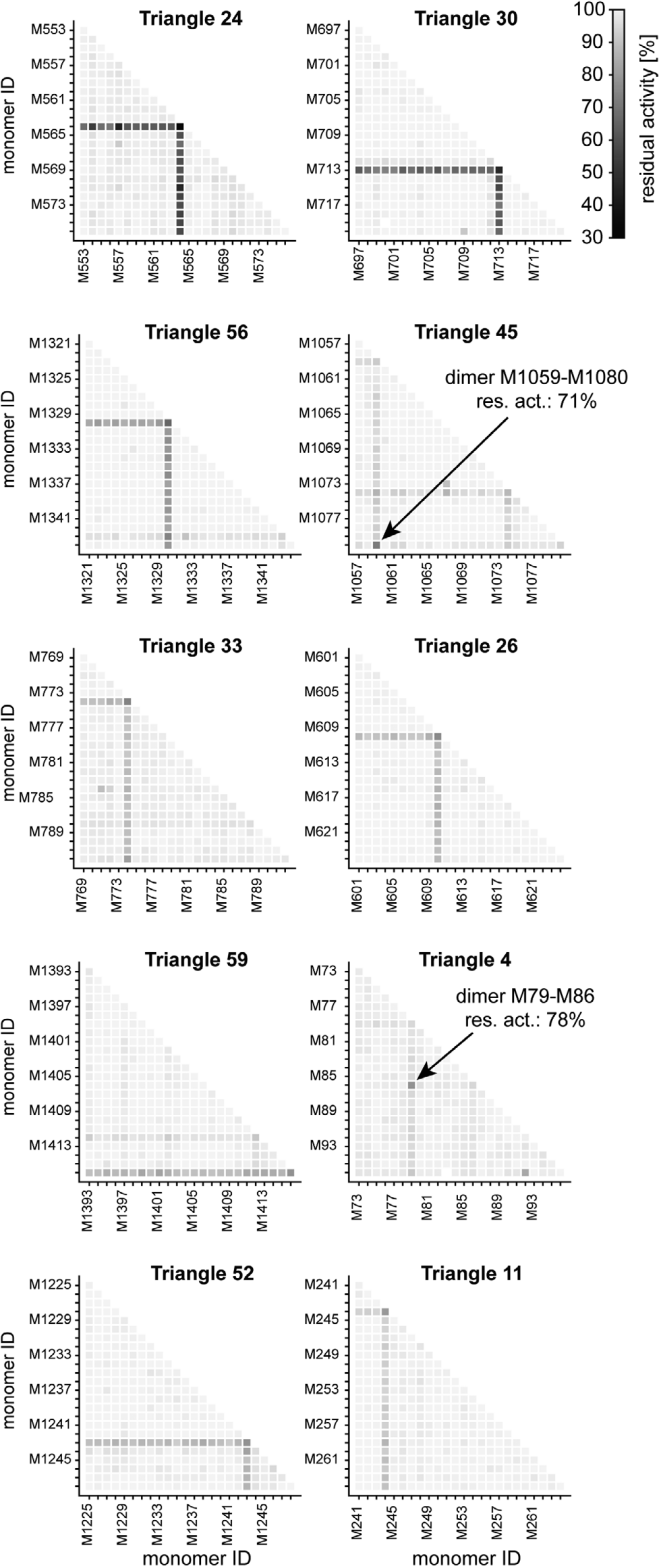
Hits of Library 1 screened for thrombin inhibition. Ten out of the 64 triangles that show hits with the highest inhibitory activity are shown. Residual activity of thrombin is indicated in greyscale. The heterodimer concentration in the screen was around 1 µM.

### 2.4 Identification of Thrombin‐Inhibiting Peptide Pairs

The strongest thrombin inhibition activity of 83% was observed for heterodimer M30‐M44 in group 2 (triangle 2), which is shown in Figure 3 (17% residual activity). More hits with strong inhibition activity (best hit > 20% thrombin inhibition) are shown in the groups displayed in Figure 4, with activities decreasing from the top to the bottom, and all other triangles are shown in Figure S6. For efficient structure‐activity relationship (SAR) exploration, we developed a software and an interactive heatmap that directly links the monomer structures and raw data to the thrombin inhibition activity and that we made available online (https://library1‐interactive‐heatmap.streamlit.app/). In general, two types of hits were observed, those that cluster in lines and those that are isolated dots in the heatmap. Since all dimers on a line share one of the monomers, it can be assumed that this monomer makes a dominant contribution to the binding. Some of the peptide pairs on lines show rather variable activity (dotted lines), suggesting that the second monomer contributes to binding to different extents. For example, the line of monomer M44 in triangle 2 shows a dotted line, with the heterodimer M30–M44 inhibiting thrombin best (83% inhibition; Figure 3). A second monomer that shows strong inhibition and a dotted line is M564 in triangle 24 (Figure 4). The second type of hit found were peptides that occurred “isolated” on the heatmaps, wherein the active combination appears as the only hit on an otherwise nearly inactive line. The most active such hits were M1059–M1080 in triangle 45 (29% inhibition) and M79–M86 in triangle 4 (22% inhibition; indicated with black arrows in Figure 4).

An analysis of the chemical structures of the hits showed a strong enrichment of peptides either containing arginine or a building block with a chlorothiophene moiety, both of which are known to interact with the thrombin's substrate recognition pocket S1. While the prevalence of these groups in the library is only 8.4%, they are found in 29 of the 31 hit dimers (93.5%), which inhibited thrombin by more than 40% (Figure S7). Instead of characterizing these hits found in Library 1 as purified products, we chose to move on with screening larger peptide pair libraries, planning to perform purification and characterization of potentially even better hits.

### 2.5 Screening Peptide Pairs in Pools

To screen a larger number of peptide pairs while keeping consumable costs low (e.g., screening plates, assay reagents), we proposed to screen peptide pairs as mixtures and thus multiple ones per well of a 1536‐well plate. We reasoned that the active peptide pair in a mixture could easily be identified in a deconvolution screen where monomers of a “mixture hit” are paired individually and their activity measured. Given that the peptide had only to be paired by ADE but not resynthesized by SPPS, such a deconvolution could be done in a single day. We chose a design for this second library, Library 2, where we grouped 1308 monomers into small pools of six (218 pools, P1–P218) and where such pools were mixed (Figure 5a). The total number of heterodimers produced and screened with this strategy was 854 778. We kept the general layout of a full coverage heatmap where results for each combination can be easily located by finding the two peptide monomers on the *x*‐ and *y*‐axes, but instead of a single monomer per row and column, a pool of six peptides is represented. Each square of the heatmap therefore contains a mixture of 12 monomers, six from the pool on the *x*‐axis and six from the pool on the *y*‐axis, which results in 66 different heterodimers and 12 homodimers per well (Figure 5b).

**FIGURE 5:**
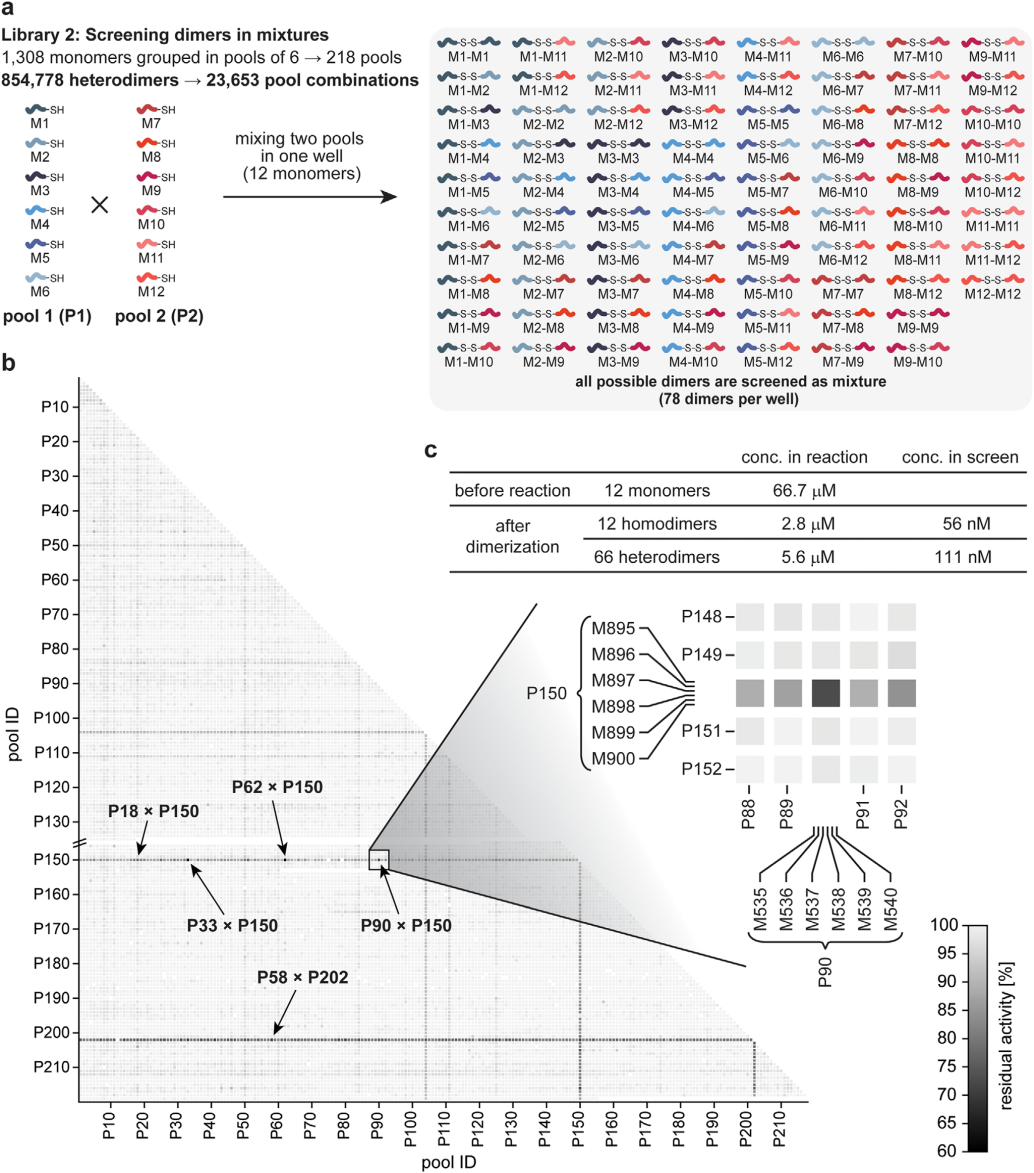
Synthesis and screening of peptide pairs in mixtures. (a) Design of dimer Library 2. Monomers M1–1308 were grouped in 218 pools (P1–P218) of 6 monomers each. In each screening well, all monomers of two pools are combined, resulting in 78 possible dimers. (b) Result of the dimer Library 2 screen for thrombin inhibition. Quantities of 10 pmol per monomer were combined by ADE. The target and assay reagents were added directly to the peptide pair mixture, resulting in a final heterodimer concentration of around 100 nM. (c) Table of concentrations of compounds in the reaction and screen.

We synthesized Library 2 using the same thiol‐peptide monomers as before and followed a similar procedure, this time transferring 10 pmol per monomer in a volume of 2.5 nL to wells of 1536‐well plates. After addition of thrombin and assay reagents in a volume of 7.5 µL, the final concentration of each heterodimer was around 100 nM (Figure 5c), meaning that only rather good inhibitors would be detected. During the screening process, we lost one microtiter plate, which reduced the number of totally analyzed peptide pairs to around 800 000 (see raw data file). Presentation of the residual thrombin activity in each well in a heatmap showed again vertical and horizontal lines that pointed to monomers that contributed particularly strongly to inhibition—as already seen in the Library 1 screen—but this time monomers in mixtures with 11 other monomers (Figure 5b). The two strongest lines in the heatmap were seen for mixtures containing either pool P150 or P202. These two pools contained the monomers M899 and M1208, respectively, which are the same peptides as the monomers M564 and M44 of Library 1 (we used different numbering for the monomers in the two libraries), which represented the strongest (M1208/M44) and second strongest (M899/M564) lines in the Library 1 screen. Despite the rather low concentration of each heterodimer in the Library 2 screen (around 100 nM vs. 1 µM in Library 1), some of the wells showed substantial inhibition of up to 40%. We also presented these results in an interactive heatmap, which allows for easy exploration of the SAR (https://library2‐heatmap.streamlit.app/).

### 2.6 Deconvolution to Identify Active Peptide Pairs

To identify the source of activity in the top hit pool combinations of Library 2, we performed a deconvolution synthesis and screening cycle as follows. We newly paired the peptides, but this time mixed only two monomers per well instead of two pools of six monomers. For each pool hit, we prepared all the 66 possible heterodimer combinations and the 12 homodimer combinations. We transferred by ADE quantities of 2.5 pmol per monomer that resulted in screening concentrations of around 170 nM heterodimer (these mixtures also contained 83 nM homodimer), which were similar to the concentrations in the Library 2 screen (Table S1). In controls containing only one monomer, the screening concentration of the corresponding homodimer was 330 nM. We performed the deconvolution for 16 pool combinations, most of them picked from the two strong lines of pools P150 and P202 (Figure 6 and Figure S8). The deconvolution unambiguously identified the active species of the pool screen, in most cases showing a line formed by 11 highly active peptide heterodimers and one homodimer. For the pools P150 and P202, the active monomers were confirmed to be M899 and M1208, respectively. The most active heterodimers formed based on these monomers showed around 20% thrombin inhibition, which was in the range expected based on the initial screen.

**FIGURE 6:**
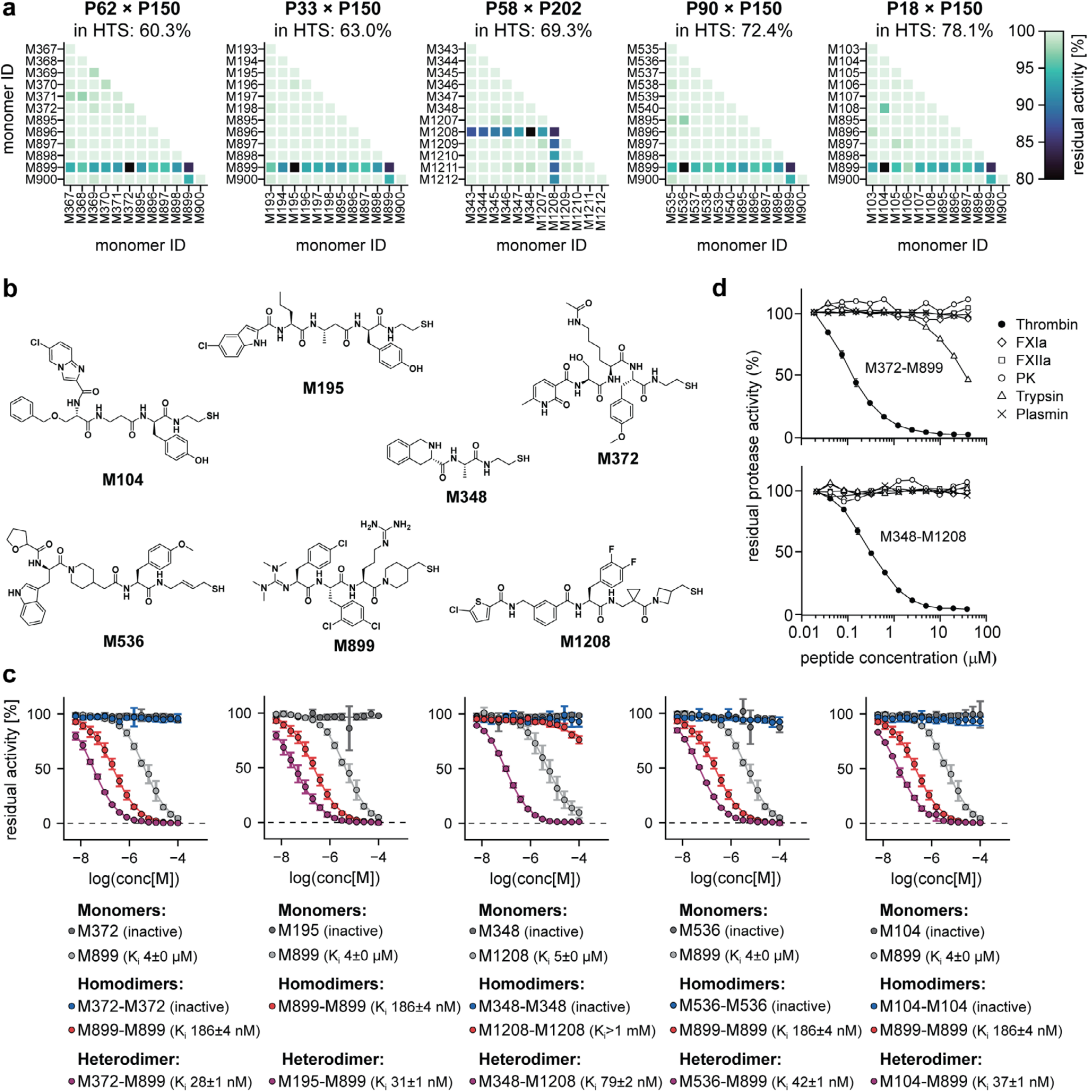
Deconvolution of pool pairs and characterization of purified peptide pairs. (a) Deconvolution screen for the five best hits identified by screening Library 2 (indicated with arrows in Figure 5). One monomer pair per well was dimerized by oxidation and screened at a heterodimer concentration of around 170 nM. (b) Chemical structures of monomers making up the most potent hits identified from Library 2 that were selected for resynthesis. The indicated monomers M348 and M899 are truncated variants of the initial peptide sequence designs. (c) Dose response curves and calculated K_i_s of purified hit heterodimers, and the corresponding homodimers and monomers. Monomers were tested under reducing conditions to prevent oxidation of the free thiol. Mean values and standard error deviations of at least three measurements (technical replicates) are indicated. (d) Specificity profiling of two peptide pairs by testing the inhibition of structurally similar trypsin‐like serine proteases.

### 2.7 Thrombin Inhibitors With Nanomolar Inhibition Constants

We next purified and characterized the five most active peptide pairs (Figure 6a). We first analyzed by LC‐MS the peptide monomers that we had produced by high‐throughput peptide synthesis as crude products and that were not all quality‐checked by mass spectrometry prior to the dimerization and HTS (Figure S9). The monomers M104, M195, M372, M536, and M1208 contained one major peptidic species with the mass of the desired peptide. For monomer M899, we found that it lacked the N‐terminal carboxylic acid building block and that it formed with its N‐terminal amine a guanidinium adduct instead. For monomer M348, we found that the N‐terminal two building blocks were truncated and that it had a free N‐terminus. For characterization, we purified the monomers, heterodimers, and homodimers of the five peptide pairs (Figure S10). For molecules involving the truncated M899 and M348, we produced and purified the truncated species that were present in the initial Library 2 screen and the deconvolution screen (Figure 6b). The heterodimeric peptide pairs and their corresponding homodimeric by‐products exhibited distinct retention times during preparative reversed‐phase HPLC, allowing straightforward separation and isolation of the heterodimers in high purity. Following purification, no detectable disulfide exchange was observed, enabling the biological activity to be determined for single, well‐defined molecular species.

Pleasingly, the purified heterodimer showed strong thrombin inhibition activity with inhibitory constants (K_i_s) in the double‐digit nanomolar range. As expected, the heterodimers were the most active species, followed by the homodimer of the dominantly active monomer (Figure 6c). The second, less active monomers and their homodimers were essentially inactive (Figures S11 and S12). The heterodimer M372‐M899 was the most potent inhibitor with a *K*_i_ of 28 ± 1 nM. Monomer M899 inhibited thrombin with a *K*_i_ of 4 ± 0 µM, showing that appending the monomer M372 improves its activity > 100‐fold. A similar result was found for the peptide pair M348‐M1208 that is based on two completely different monomers and had a *K*_i_ of 80 ± 2 nM which is 62‐fold better than the more potent one of the two monomers, M1208 (*K*_i_ of 5 ± 0 µM). A specificity profiling of the peptide pairs M372‐M899 and M348‐M1208 revealed that the inhibitors bind with high selectivity, not inhibiting the physiologically important plasma proteases Factor XIa, Factor XIIa, plasma kallikrein, and plasmin, despite the high sequence and structure similarity of these trypsin‐like serine proteases (Figure 6d). One of the peptide pairs, M372–M899, inhibited the digestive protease trypsin but only at high concentrations. Taken together, the characterization of purified dimers and monomers showed that the new strategy yielded, as anticipated, large affinity gains by combining peptide fragments, and that the inhibitors display an excellent target selectivity.

## 3 Conclusions

We developed a strategy to produce and test a large number of nearly one million chemically synthesized peptides in microtiter plates. The key to achieving such a large number was that we did not produce the peptides as whole sequences by solid‐phase synthesis but as short, “half‐peptides” that were combinatorially ligated to desired‐length “whole‐peptides” in a solution‐phase reaction. In contrast to SPPS, which is currently limited to 384‐well plates and requires a relatively large synthesis scale of around 1 micromole, the ligation of short peptides could be carried out using acoustic liquid transfer in 1536‐well plates and on a small scale of a few picomoles and thus with over 10 000 times less material used per library member.

In this study, multiple peptide monomers were combined in each well to enable the screening of large numbers of peptide pairs while minimizing consumable costs, with the 1,536‐well microplates representing the largest expense (approximately US$30 per plate). However, screening mixtures reduces the concentration of each individual peptide pair, which may limit the identification of ligands for more challenging targets where only weak or moderate‐affinity binders are present in the library. For such targets, screening a single peptide pair per well at a conventional HTS concentration (e.g., 10 µM) may be a more effective strategy. In this format, an 800 000‐member peptide‐pair library would require approximately 520 microwell plates, which remains economically feasible. Due to the fast reagent transfer by an acoustic dispenser, the library synthesis and screening could be done in around a week.

By using disulfide bridge formation as a pairing reaction, we were able to efficiently ligate the peptides in nanoliter volumes and under mild conditions, which allowed the products to be tested unpurified in the same microwell plates that were used for the ligation reaction. By combining multiple peptides per well and an efficient deconvolution strategy, we were able to massively increase the number of peptides produced and tested to over 800 000. A limitation of the developed peptide‐pairing strategy, which relies on ligation through two thiol groups, is the formation of homodimeric side products. This limitation could potentially be overcome by employing two orthogonal reactive groups, such as azide and alkyne for click chemistry or amine and carboxylic acid for amide bond formation. These approaches would enable directional coupling and selectively generate the desired heterodimeric products while preventing homodimer formation. Furthermore, the resulting linkage would be redox stable and could potentially be retained throughout the drug development process without requiring further optimization.

In contrast to biological display methods such as phage or mRNA display, which can be used to screen even much larger peptide libraries (over a billion peptides), the method presented here allows the incorporation of an almost infinite number of chemically and structurally highly diverse building blocks, such as hundreds of amino acids and thousands of carboxylic acids. This makes it possible to achieve a large diversity and library size with relatively short peptides having a molecular weight of around 800 to 1500 Da, which is important if membrane permeability is required. We did not experimentally assess the membrane permeability of the thrombin inhibitors identified in this study, and additional optimization may be required to confer cell permeability, as well as to improve other drug‐like properties such as proteolytic or metabolic stability. Nevertheless, peptides with molecular weights below 1 kDa and a limited polar surface area have in the past provided promising starting points for the development of cell‐permeable and/or orally bioavailable peptidomimetic drugs.

Another important advantage of the method presented here is that the synthetic peptide libraries are arrayed in microtiter plates, which allows them to be tested using a wide range of different functional assays. In contrast to methods based on affinity selections, functional assays provide molecules that bind to desired sites of the protein target and have the desired biological effect. This helped us in this work to identify several nanomolar inhibitors of the relatively easy proof‐of‐concept disease target thrombin. We are optimistic that the presented method is applicable to other targets and potentially also to more challenging ones.

## Author Contributions

**Anne Zarda**: conceptualization, investigation, writing – original draft, methodology, validation, visualization, writing – review and editing, software, formal analysis, data curation, supervision. **Xingwang Deng**: methodology, validation, conceptualization, investigation. **Edward Will**: investigation, validation, methodology, writing – review and editing, formal analysis. **Thomas Emel**: methodology, visualization, writing – review and editing, investigation. **Maylin Romero**: methodology, investigation, validation. **Christian Heinis**: conceptualization, investigation, funding acquisition, writing – review and editing, visualization, validation, formal analysis, project administration, data curation, supervision, resources.

## Conflicts of Interest

Christian Heinis is a co‐founder of Orbis Medicines. All other authors declare no competing interest.

## Supporting information




**Supporting File 1**: Supplementary tables, figures, compound characterization data, and screening results. The code for the interactive heatmaps is available on GitHub or hosted as web applications on Streamlit:
https://github.com/mtzknd/library1_interactive_heatmap
https://github.com/mtzknd/library2_interactive_heatmap
https://library1‐interactive‐heatmap.streamlit.app/
https://library2‐heatmap.streamlit.app/).

**Supporting File 2**: anie74579‐sup‐0002‐SuppMat.pdf.

## Data Availability

The data that supports the findings of this study are available in the supplementary material of this article.
