# Supplementary material for: Chemical Synthesis and Functional Screening of an 800 000‐Member Library Using a Peptide‐Pairing Strategy
Source: Angew Chem Int Ed Engl. 2026 Sep 8;65(44):e3693802. doi: 10.1002/anie.3693802 (PMC13703915; doi:10.1002/anie.3693802)
Supplement: Supplementary file 1 [file ANIE-65-e3693802-s002.pdf]

Supporting Information

Chemical Synthesis and Functional Screening of an 800,000-Member Library Using a Peptide-Pairing Strategy

Anne Zarda,[1] Xingwang Deng,[1] Edward Will,[1] Thomas Emel,[1] Maylin Romero[1] and Christian Heinis[1,*]

[a]Institute of Chemical Sciences and Engineering, School of Basic Sciences, École Polytechnique Fédérale de Lausanne (EPFL), CH-1015 Lausanne, Switzerland.

*Corresponding authors: christian.heinis@epfl.ch

MATRIALS AND METHODS

Liquid chromatography-mass spectrometry (LC-MS) analysis

LC-MS analysis was conducted on a LCMS 2020 single quadrupole liquid chromatography mass spectrometer (Shimadzu) with a C18 reversed phase (RP) column (Kinetex 2.6 µm C18 100 Å, LC Column 50×2.1 mm, Phenomenex). Ionization was achieved by electrospray ionization. Different gradients of acetonitrile (ACN, 0.05% formic acid) in water (0.05% formic acid) were applied at a flow rate of 1 mL/min.

Automated solid-phase peptide synthesis (SPPS) of thiol peptides

To obtain peptides with thiol groups at the C-terminal end, we applied a solid support carrying a disulfide linker so that a thiol-functionalized peptide could be released by disulfide reduction. For variation of the C-terminal thiol-containing building block, a method for the preparation of disulfide-linked cysteamine derivatives was used.[30] The resins with disulfide-linked cysteamine derivatives were stored at −20 °C until use.

In syringes

Peptides were synthesized in 5 mL reactors for SPPS (Roth Selection, catalog number 7927) on a MultiPep 2 RSi peptide synthesizer (Intavis). Resins carrying the desired cysteamine building block, for a synthesis scale of 50 µmol (or less in some experiments), were added to the 5 mL reactors and washed 3× with 2 mL N,N-dimethylformamide (DMF). For each coupling, 530 µL Fmoc-amino acid or carboxylic acid (500 mM in N-methylpyrrolidone, NMP, 265 µmol), 125 µL N-methylmorpholine (NMM, 4 M in NMP, 500 µmol) and 500 µL 1-[bis(dimethylamino)methylene]-1H-1,2,3-triazolo[4,5-b]pyridinium 3-oxide hexafluorophosphate (HATU, 500 mM, 250 µmol) were pre-mixed for 1 min before addition to the resins. The reactors were agitated during the 1 h incubation period. The final concentrations in the coupling reaction were 229 mM derivative, 432 mM NMM and 216 mM HATU. The reaction was performed twice, and resins were washed 3× with 2 mL DMF. Subsequently, the Fmoc-protecting group was removed with 900 µL 20% piperidine in DMF allowing a reaction time of 2 min. Deprotection was performed twice before washing 6× with 2 mL DMF.

In 96-well plates

Peptides were synthesized in 96-well filter plates (Orochem, catalog number: OF 1100) on a MultiPep 2 RSi peptide synthesizer (Intavis). Resins carrying the desired cysteamine building block, for a synthesis scale of around 5 µmol, were added to the wells of fritted 96-well plate and washed 3× with 225 µL DMF. For each coupling, 56 µL of derivative (Fmoc-amino acid or carboxylic acid, 500 mM in NMP, 28 µmol), 13 µL NMM (4 M in NMP, 52 µmol) and 52 µL HATU (500 mM in NMP, 26 µmol) were pre-mixed for 1 min before addition to the resins. The final concentrations in the coupling reaction were 231 mM

derivative, 430 mM NMM and 215 mM HATU. The reaction was performed for 1 h twice, and resins were washed 3× with 250 µL DMF. Subsequently, the Fmoc-protecting group was removed with 120 µL 20% piperidine in DMF allowing a reaction time of 2 min. Deprotection was performed twice before washing 6× with 225 µL DMF.

In 384-well plates

Peptides were synthesized in 384-well filter plates (Agilent Technologies, catalog number: 201035-100) on a MultiPep 2 RSi peptide synthesizer (Intavis) to enable the synthesis of 1,536 peptides in parallel (four 384-well reactor plates). Resins carrying the desired cysteamine building block were distributed to the synthesis plates for a synthesis scale of around 1 µmol using a resin dispenser tool. At the beginning of the synthesis, resins were washed 2× with 70 µL DMF. For each coupling, 13 µL of Fmoc-amino acid or carboxylic acid, 500 mM in NMP (6.5 µmol, 6.5 eq), 3 µL NMP, 4 µL NMM (4 M in NMP, 16 µmol, 16 eq) and 12 µL HATU (500 mM in NMP, 6 µmol, 6 eq) were pre-mixed for 1 min before addition to the resin. Remark: when removing the amino acid tubes from the synthesizer, we observed in some of them precipitates at the bottom, indicating that a concentration of 500 mM was too high and could be optimized in future. After 1 h, 31 µL NMP were added to each well and incubated for 1 h. The step of adding 31 µL NMP after one hour was introduced to first allow reaction at a high reagent concentration (1st hour) and to ensure full coverage of all resin with the larger solvent volume (2nd hour). The final concentrations in the coupling reaction were 203 mM or 103 mM derivative, 500 mM or 254 mM NMM and 187.5 mM or 95.2 mM HATU before and after NMP addition, respectively. Coupling was performed twice, and resins were washed 6× with 70 µL DMF. Subsequently, the Fmoc-protecting group was removed with 35 µL 20% piperidine in DMF allowing a reaction time of 2 min. Deprotection was performed twice before washing 8× with 70 µL DMF.

Side chain deprotection and reductive release procedure

In syringes

Acid labile protecting groups were removed by incubation of resins with 2 mL deprotection solution (95% trifluoroacetic acid, TFA, 2.5% triisopropylsilane, TIPS, 2.5% H_2O) for 1 h twice. Resins were washed 3× with 2 mL dichloromethane (DCM) and dried (between 1 h and overnight on vacuum). A final wash using 3× 2 mL DMF was conducted before the addition of 2 mL release solution (100 mM butanedithiol, BDT, 100 mM triethylamine, TEA in DMF). Release was conducted twice with incubation times of 4 h and overnight respectively.

In 96-well plates

Peptides were released from the resin with 200 µL release solution (100 mM BDT, 100 mM TEA in DMF). Release was conducted twice with incubation times of 5 h and overnight respectively. Released peptides

were collected via centrifugation into 96-well deep well plates (Fisherbrand, catalog number: 12566121) and stored at −20 °C.

In 384-well plates

Acid labile protecting groups were removed by incubation of resins with 50 µL deprotection solution (95% TFA, 2.5% TIPS, 2.5% H_2O) for 2 h twice. Resins were washed 3× with 50 µL DCM and dried (between 1 h and overnight) before addition of 60 µL release solution (67 mM BDT, 67 mM TEA in DMF). Release was conducted twice with incubation times of 3 h and overnight respectively. Released peptides were collected via centrifugation into 384-well deep well plates (Corning, catalog number: 3347) and stored at −20 °C.

Preparation of thiol peptide stocks in dimethyl sulfoxide (DMSO)

For purified model peptides

The release solution containing the peptides was acidified using 615 µL 10% TFA in H_2O and peptides were dried by rotational vacuum concentration (RVC) using an RVC 2-33 CDplus with an Alpha 2-4 LSCbasic freeze drying unit (Martin Christ Freeze Dryers). The peptide crudes were purified using a RP-HPLC system (2489 UV/visible detector, 2535 quaternary gradient module, fraction collector III, Waters) with a C18 columns (Xterra Prep MS C18 OBD 10 µm, 19×250 mm column, Waters, catalog number: 186002259). Purified peptides were lyophilized and 100 mM stock solutions in DMSO were prepared.

For crude model peptides

Peptides were transferred to ECHO qualified 384-well PP 2.0 microplates (Beckman Coulter, formerly Labcyte; catalog number: PP-0200). Release solution was acidified with 61 µL 10% TFA in H_2O and solvent was evaporated by RVC. Peptides were dissolved in 50 µL DMSO and stored at −20 °C.

For monomer library

Peptides were transferred to ECHO qualified 384-well PP 2.0 microplates (Beckman Coulter, formerly Labcyte; catalog number: PP-0200). Release solution was evaporated by RVC. Peptides were re-reduced overnight with 40 µL reducing solution (100 mM BDT, 100 mM TEA in DMF). The solutions were acidified with 7 µL 10% TFA in H_2O (9.1 µmol) and solvent was removed by RVC. Peptides were dissolved in 40 µL DMSO and stored at −20 °C.

Thiolpeptide concentration determination

The concentration of crude thiol peptides was determined via Ellman's assay. A volume of 135 nL of thiol-peptide was transferred to black 384-well plates with transparent bottom (Greiner, catalog number: 781096) using acoustic droplet ejection on an ECHO650 system (Beckman Coulter, formerly Labcyte) and 23.7 µL of assay buffer (120 mM NH_4HCO_3, 90% H_2O, 10% DMSO, pH 8) were added by liquid bulk

dispensing using CERTUS Flex (Gyger). Also with liquid bulk dispensing, 6 µL 10 mM dithionitrobenzoic acid (DTNB) in buffer (60 nmol) were added and plates were centrifuged before measuring absorbance at 412 nm using an infinite M200 PRO plate reader (TECAN). Measured values were compared to a calibration curve prepared with cysteine dilutions (for model peptides: 100–0.5 mM, for library: 50–0.8 mM; 2-fold dilutions) of which also 135 nL volumes were transferred.

Preparation of peptide stocks with adjusted concentration

For library generation

Using the previously determined concentrations, 5 nmol of each peptide were transferred via acoustic droplet ejection to ECHO qualified 1536-well low dead volume microwell plates (Beckman Coulter, formerly Labcyte, catalog number: LP-0400) and DMSO was evaporated by RVC. For the preparation of a 4 mM stock, the step of transferring 5 nmol and solvent evaporation was repeated three times. Oxidized thiol groups were reduced by adding 2.5 µL reducing solution (100 mM BDT, 100 mM TEA in DMF) and incubation for 3 h. Samples were acidified with 2 µL 10% TFA in water and dried by RVC. Peptides were dissolved in DMSO to obtain stocks of 1 mM (for Library 1) or 4 mM (for Library 2). The DMSO stocks were stored at −20 °C to prevent rapid oxidation and used within six months.

For model peptides

Peptide stocks were diluted with DMSO to a final peptide concentration of 1 mM.

Dimerization procedure

For model reactions

Peptide monomers in DMSO were transferred to an ECHO qualified 384-well PP 2.0 microplate using volumes described in Table S1. Dimerization buffer composed of NH_4HCO_3 in H_2O and DMSO was added by pipetting using the volume indicated in Table S1 (final concentration: 60 mM NH_4HCO_3, 50% H_2O, 50% DMSO). Reaction mixtures were incubated for 24 h at room temperature (rt). The reaction mixture was analyzed by LC-MS wherein the samples were prepared by adding 4 µL reaction mixture to 16 µL H_2O:ACN [1:1] and 1 µL was injected.

For library generation

All plate handling was done by the robotic arm of an integrated screening platform (Access laboratory workstation, Beckman Coulter, formerly Labcyte). Peptide monomers were transferred to black 1,536-well OptiPlates (Perkin Elmer, catalog number: 6004270) using acoustic droplet ejection. The applied volumes and concentrations are shown in Table S1. Subsequently, 75 nL dimerization buffer (120 mM NH_4HCO_3 in H_2O) and 45 nL DMSO were added to reach a final volume of 150 nL using liquid bulk dispensing. Plates were spun for 2 min at 800 g (2,500 rpm in a rotor with a radius of 11 cm). Reaction

mixtures were covered with MicroClime environmental microplate lids (Beckman Coulter, formerly Labcyte, catalog number: LL-0310) and incubated for 24 h at rt.

For deconvolution screen

Peptide monomers were transferred to black 1,536-well OptiPlates (Perkin Elmer, catalog number: 6004270) using acoustic droplet ejection. The applied volumes and concentrations are shown in Table S1. Subsequently, 75 nL dimerization buffer (120 mM NH_4HCO_3 in H_2O) and 70 nL DMSO were added to reach a final volume of 150 nL using liquid bulk dispensing. Plates were spun for 2 min at 800 g (2,500 rpm in a rotor with a radius of 11 cm). Reaction mixtures were covered with MicroClime environmental microplate lids (Beckman Coulter, formerly Labcyte, catalog number: LL-0310) and incubated for 24 h at rt.

For hit characterization

After release from the resin, the cleavage solvent and reagents were evaporated using RVC, and the peptides purified using a RP-HPLC system (2535 quaternary gradient module, 2489 UV/visible detector, and fraction collector III, Waters) with a C18 column (Xterra Prep MS C18 OBD 10 μm, 19×250 mm column, Waters, catalog number: 186002259). Purified monomers were lyophilized and dissolved in DMSO to prepare 100 mM stock solutions. Then, equal amounts of two monomers were combined and 120 mM NH_4HCO_3 buffer was added to dilute the solution to 50% DMSO. If precipitation occurred, DMSO was added stepwise until the peptides were fully dissolved. Dimerization reactions were monitored by LC-MS analysis of samples, preparing samples by diluting 2 μL of reaction mixture in 18 μL H_2O:ACN [1:1]. After completion of the dimerization reaction, the dimers were purified using an Ultimate 3000 UHPLC (Thermo Scientific) with a C18 column (NovaPak CSH 5 μm, 10×150 mm column, Waters, catalog number: 186008238) and a solvent system of adapted gradients of buffer A (H_2O with 0.1% TFA) to buffer B (ACN with 0.1% TFA). Samples were prepared by diluting the reaction mixtures to up to 1 mL with water and acetonitrile. Purified dimers were lyophilized and dissolved in DMSO to obtain 10 mM stock solutions.

High throughput thrombin inhibition screening

All plate handling was done by the robotic arm of an integrated screening platform described above. Thrombin activity was quantified using the fluorogenic substrate Cbz-G-G-R-AMC (Bachem, catalog number: 4002155) and measuring fluorescence intensity after different time points. A volume of 3.7 μL of 8.2 nM human thrombin (Innovative Research, cat. no #IHUTHRA) in assay buffer (50 mM Tris-HCl, 150 mM NaCl, 10 mM $MgCl_2$, 1 mM $CaCl_2$, 0.01 % Triton-X, 0.1 % bovine serum albumin (BSA), pH 7.4) was added to the crude peptide dimerization reactions in 1536-well OptiPlates using bulk dispensing and incubated for 10 min. To each well, 3.7 μL 102 μM Cbz-G-G-R-AMC in assay buffer with 0.1% DMSO was added by liquid bulk dispensing, and the plates were centrifuged (800 g, 2 min; 2,500 rpm, rotor

radius of 11 cm). Final thrombin concentration: 4 nM, final substrate concentration: 50 µM, final DMSO concentration: 1.1%, final NH_4HCO_3 concentration: 1.2 mM, final peptide dimer concentrations are listed in Table S1. Fluorescence intensity was measured (excitation wavelength: 384 nm, emission wavelength: 440 nm) in time increments of 150 s over 7 cycles using a PHERAstar FSX plate reader (BMG Labtech). The fluorescence intensity increase over time was fitted using linear regression. Slopes were normalized to an average of 48 negative controls (no monomers, no peptide dimers) measured on the same assay plate. The Pearson coefficient of correlation (rvalue) was calculated for each well. Data of wells showing an rvalue lower than 0.998 or a fluorescence intensity at the first time point higher than 40,000 units were excluded from the analysis.

Interactive heatmaps

Screening results for Library 1 and 2 were made available as an interactive heatmap for easy data exploration. The code is open accessible on github (Library 1: https://github.com/mtzknd/library1_interactive_heatmap, Library 2: https://github.com/mtzknd/library2_interactive_heatmap) to be run on a local machine or as an web application hosted by streamlit (Library 1: https://library1-interactive-heatmap.streamlit.app/, Library 2: https://library2-heatmap.streamlit.app/).

Half maximal inhibitor concentration (IC_{50}) and inhibitory constant (K_i) determination

To determine IC_{50} values of purified compounds, 2-fold serial dilutions of compound were prepared in assay buffer (50 mM Tris-HCl, 150 mM NaCl, 10 mM $MgCl_2$, 1 mM $CaCl_2$, 0.01% [v/v] Triton-X, 0.1% BSA, pH 7.5) containing 3% DMSO, starting with 300 µM as the highest concentration. A volume of 5 µL of each dilution was transferred to 384-well shallow well plates (Thermo Scientific Nunc, catalog number: 264705) and 5 µL of thrombin (12 nM) in assay buffer was added using liquid bulk dispensing. After a 10-minute incubation period, 5 µL of substrate in assay buffer (150 µM) containing 0.15% DMSO were added by liquid bulk dispensing, and the plates were centrifuged (480 g, 2 min; 1,500 rpm, rotor radius of 19.2 cm). Final thrombin concentration: 4 nM, final substrate concentration: 50 µM, final DMSO concentration: 1.1%. Fluorescence intensity (excitation wavelength: 384 nm, emission wavelength: 440 nm) was measured on a PHERAstar FSX plate reader (BMG Labtech) in time increments of 150 s over 22.5 minutes. The fluorescence intensity increase over time was fitted using linear regression. Slopes were normalized to an average of negative controls (no monomers, no peptide dimers) measured on the same plate (n>10). IC_{50} determination under reducing conditions was performed by applying the same conditions but adding tris(2-carboxyethyl)phosphine hydrochloride (TCEP-HCl) to the assay buffer to reach a concentration of 1 mM.

Specificity profiling

Protease selectivity was evaluated by measuring the inhibition of a panel of trypsin-like serine proteases using fluorogenic substrate assays. Peptide inhibitors were tested as twofold serial dilutions covering a concentration range from 40 µM to 19.5 nM. The final enzyme concentrations were 2.2 nM human thrombin (Molecular Innovations), 5 nM human plasma kallikrein (Innovative Research), 1 nM human factor XIa (Innovative Research), 0.2 nM bovine trypsin (Molecular Innovations), 5 nM human factor XIIa (Molecular Innovations), and 1 nM human Lys-plasmin (Innovative Research). Fluorogenic substrates were used at a final concentration of 50 µM: Z-Gly-Gly-Arg-AMC (Bachem) for thrombin and trypsin, Z-Phe-Arg-AMC (Bachem) for plasma kallikrein, Boc-Phe-Ser-Arg-AMC (Bachem) for factor XIa, Boc-Gln-Gly-Arg-AMC (Bachem) for factor XIIa, and D-Val-Leu-Lys-AMC (Bachem) for plasmin. Assays were carried out in black 96-well microplates with a total reaction volume of 150 µL. First, 50 µL of each peptide dilution prepared in assay buffer (50 mM Tris-HCl, 150 mM NaCl, 10 mM $MgCl_2$, 1 mM $CaCl_2$, 0.01% [v/v] Triton-X, 0.1% BSA, pH 7.4) containing 1.2% DMSO, was added to the wells, followed by 50 µL of the corresponding protease solution. After a 10 min preincubation at 25 °C, the reactions were initiated by adding 50 µL of substrate solution in assay buffer containing 1.8% DMSO. Fluorescence intensity (excitation wavelength: 384 nm, emission wavelength: 440 nm) was measured on a PHERAstar FSX plate reader (BMG Labtech) in time increments of 150 s over 22.5 minutes. The fluorescence intensity increase over time was fitted using linear regression. Slopes were normalized to the lowest peptide concentration well, measured on the same plate.

SUPPLEMENTARY TABLES

Table S1. Volumes and concentrations of monomers applied in different dimerization libraries.

	Model reaction with purified peptide	Model reaction with crude peptide	Library 1	Library 2	Library 2 hit deconvolution
**Number of different monomers per well**	2	2	2<br>(1 in controls)	12	2<br>(1 in controls)
**c (monomer stock) [mM]**	10	1	1	4	1
**V (transferred monomer) [nL]**	1,000	2,000	15<br>(30 in controls)	2.5	2.5<br>(5 in controls)
**V (reaction total) [nL]**	100,000	20,000	150	150	150
**c (each monomer in reaction) [μM]**	100	100	100	66.7	16.7<br>(33.3 in controls)
**c (each monomer in screen/assay) [μM]**	N/A	N/A	2	1.3	0.33
**c (each heterodimer in screen/assay) [μM]**	N/A	N/A	1<br>(2 in controls)	0.11	0.17

Table S2. Aminothiol building blocks used for peptide synthesis. The building blocks were immobilized on polystyrene solid phase via a disulfide bridge.[30]

Abbreviation	Number in reference[30]
**AT1**	1
**AT2**	4
**AT3**	7
**AT4**	5

Table S3. Amino acids and carboxylic acids used for the synthesis of library peptides.

Abbreviation	CAS number	IUPAC name
**X1**	135112-27-5	(2S)-2-(9H-fluoren-9-ylmethoxycarbonylamino)butanoic acid
**X2**	135112-28-6	(2S)-2-(9H-fluoren-9-ylmethoxycarbonylamino)pentanoic acid
**X3**	143688-83-9	(2R,3R)-2-(9H-fluoren-9-ylmethoxycarbonylamino)-3-methylpentanoic acid
**X4**	114360-54-2	(2R)-2-(9H-fluoren-9-ylmethoxycarbonylamino)-4-methylpentanoic acid
**X5**	35661-60-0	(2S)-2-(9H-fluoren-9-ylmethoxycarbonylamino)-4-methylpentanoic acid
**X6**	198561-07-8	(2S)-2-(9H-fluoren-9-ylmethoxycarbonylamino)pent-4-ynoic acid
**X7**	214750-76-2	(2S)-3-cyclopropyl-2-(9H-fluoren-9-ylmethoxycarbonylamino)propanoic acid
**X8**	84000-07-7	(2S)-2-[9H-fluoren-9-ylmethoxycarbonyl(methyl)amino]propanoic acid
**X9**	252049-05-1	(2S)-2-[9H-fluoren-9-ylmethoxycarbonyl(methyl)amino]pentanoic acid
**X10**	112883-42-8	(2S)-2-[9H-fluoren-9-ylmethoxycarbonyl(methyl)amino]hexanoic acid
**X11**	218926-46-6	2-ethyl-2-(9H-fluoren-9-ylmethoxycarbonylamino)butanoic acid
**X12**	126705-22-4	1-(9H-fluoren-9-ylmethoxycarbonylamino)cyclopropane-1-carboxylic acid
**X13**	136552-06-2	(2S)-1-(9H-fluoren-9-ylmethoxycarbonyl)azetidine-2-carboxylic acid
**X14**	117322-30-2	1-(9H-fluoren-9-ylmethoxycarbonylamino)cyclopentane-1-carboxylic acid
**X15**	193693-60-6	2-[(2S)-1-(9H-fluoren-9-ylmethoxycarbonyl)pyrrolidin-2-yl]acetic acid
**X16**	86069-86-5	(2S)-1-(9H-fluoren-9-ylmethoxycarbonyl)piperidine-2-carboxylic acid
**X17**	193693-68-4	(3S)-1-(9H-fluoren-9-ylmethoxycarbonyl)piperidine-3-carboxylic acid
**X18**	162648-54-6	1-(9H-fluoren-9-ylmethoxycarbonylamino)cyclohexane-1-carboxylic acid
**X19**	381241-08-3	(1R,2R)-2-(9H-fluoren-9-ylmethoxycarbonylamino)cyclohexane-1-carboxylic acid
**X20**	167690-53-1	trans-4-(9H-fluoren-9-ylmethoxycarbonylamino)methylcyclohexanecarboxylic acid
**X21**	1217675-84-7	cis-4-(9H-fluoren-9-ylmethoxycarbonylamino)cyclohexaneacetic acid
**X22**	1217650-00-4	trans-4-(9H-fluoren-9-ylmethoxycarbonylamino)cyclohexaneacetic acid

**X23**	214750-71-7	(2S)-1-(9H-fluoren-9-ylmethoxycarbonyl)-2,3,3a,4,5,6,7,7a-octahydroindole-2-carboxylic acid
**X24**	173212-86-7	(2S)-2-(9H-fluoren-9-ylmethoxycarbonylamino)-4-methoxybutanoic acid
**X25**	128107-47-1	(2R)-2-(9H-fluoren-9-ylmethoxycarbonylamino)-3-[(2-methylpropan-2-yl)oxy]propanoic acid
**X26**	71989-33-8	(2S)-2-(9H-fluoren-9-ylmethoxycarbonylamino)-3-[(2-methylpropan-2-yl)oxy]propanoic acid
**X27**	201481-37-0	(2S,3S)-2-(9H-fluoren-9-ylmethoxycarbonylamino)-3-[(2-methylpropan-2-yl)oxy]butanoic acid
**X28**	138797-71-4	(2R,3S)-2-(9H-fluoren-9-ylmethoxycarbonylamino)-3-[(2-methylpropan-2-yl)oxy]butanoic acid
**X29**	133174-15-9	(2S)-5-(carbamoylamino)-2-(9H-fluoren-9-ylmethoxycarbonylamino)pentanoic acid
**X30**	260367-12-2	2-[2-(9H-fluoren-9-ylmethoxycarbonylamino)ethoxy]acetic acid
**X31**	117106-20-4	(2S,3R)-2-[9H-fluoren-9-ylmethoxycarbonyl(methyl)amino]-3-[(2-methylpropan-2-yl)oxy]butanoic acid
**X32**	122996-47-8	(2S,4R)-1-(9H-fluoren-9-ylmethoxycarbonyl)-4-[(2-methylpropan-2-yl)oxy]pyrrolidine-2-carboxylic acid
**X33**	112883-40-6	(2R)-2-(9H-fluoren-9-ylmethoxycarbonylamino)-4-methylsulfanylbutanoic acid
**X34**	163437-14-7	(2S)-2-(9H-fluoren-9-ylmethoxycarbonylamino)-4-methylsulfonylbutanoic acid
**X35**	133054-21-4	(4R)-3-(9H-fluoren-9-ylmethoxycarbonyl)-1,3-thiazolidine-4-carboxylic acid
**X36**	111524-95-9	(2R)-2-(9H-fluoren-9-ylmethoxycarbonylamino)-2-phenylacetic acid
**X37**	35661-40-6	(2S)-2-(9H-fluoren-9-ylmethoxycarbonylamino)-3-phenylpropanoic acid
**X38**	213383-02-9	(2S)-3-(4-tert-butylphenyl)-2-(9H-fluoren-9-ylmethoxycarbonylamino)propanoic acid
**X39**	138774-93-3	(2R)-2-(9H-fluoren-9-ylmethoxycarbonylamino)-3-naphthalen-1-ylpropanoic acid
**X40**	155369-11-2	3-[(9H-fluoren-9-ylmethoxycarbonylamino)methyl]benzoic acid
**X41**	135944-07-9	2-(9H-fluoren-9-ylmethoxycarbonylamino)-1,3-dihydroindene-2-carboxylic acid
**X42**	169555-95-7	(2S)-2-(9H-fluoren-9-ylmethoxycarbonylamino)-3-pyridin-4-ylpropanoic acid
**X43**	142994-45-4	(2R)-2-(9H-fluoren-9-ylmethoxycarbonylamino)-3-pyridin-3-ylpropanoic acid
**X44**	205526-34-7	(2R)-3-(4-cyanophenyl)-2-(9H-fluoren-9-ylmethoxycarbonylamino)propanoic acid
**X45**	173963-93-4	(2S)-3-(4-cyanophenyl)-2-(9H-fluoren-9-ylmethoxycarbonylamino)propanoic acid
**X46**	81379-52-4	(2S)-2-(9H-fluoren-9-ylmethoxycarbonylamino)-3-[1-[(2-methylpropan-2-yl)oxycarbonyl]imidazol-4-yl]propanoic acid

**X47**	143824-78-6	(2S)-2-(9H-fluoren-9-ylmethoxycarbonylamino)-3-[1-[(2-methylpropan-2-yl)oxycarbonyl]indol-3-yl]propanoic acid
**X48**	163619-04-3	(2R)-2-(9H-fluoren-9-ylmethoxycarbonylamino)-3-[1-[(2-methylpropan-2-yl)oxycarbonyl]indol-3-yl]propanoic acid
**X49**	220497-85-8	(2R)-2-(9H-fluoren-9-ylmethoxycarbonylamino)-3-(furan-2-yl)propanoic acid
**X50**	71989-38-3	(2S)-2-(9H-fluoren-9-ylmethoxycarbonylamino)-3-[4-[(2-methylpropan-2-yl)oxy]phenyl]propanoic acid
**X51**	83792-48-7	(2S)-2-(9H-fluoren-9-ylmethoxycarbonylamino)-3-phenylmethoxypropanoic acid
**X52**	133373-24-7	(2S)-2-[9H-fluoren-9-ylmethoxycarbonyl(methyl)amino]-3-[4-[(2-methylpropan-2-yl)oxy]phenyl]propanoic acid
**X53**	205528-32-1	(2S)-2-(9H-fluoren-9-ylmethoxycarbonylamino)-3-(1,3-thiazol-4-yl)propanoic acid
**X54**	177966-60-8	(2S)-3-(1-benzothiophen-3-yl)-2-(9H-fluoren-9-ylmethoxycarbonylamino)propanoic acid
**X55**	169243-86-1	(2S)-2-(9H-fluoren-9-ylmethoxycarbonylamino)-3-(4-fluorophenyl)propanoic acid
**X56**	198560-41-7	(2S)-3-(2-chlorophenyl)-2-(9H-fluoren-9-ylmethoxycarbonylamino)propanoic acid
**X57**	175453-08-4	(2S)-3-(4-chlorophenyl)-2-(9H-fluoren-9-ylmethoxycarbonylamino)propanoic acid
**X58**	177966-59-5	(2S)-3-(3,4-dichlorophenyl)-2-(9H-fluoren-9-ylmethoxycarbonylamino)propanoic acid
**X59**	908847-42-7	(2S)-3-(6-chloro-1H-indol-3-yl)-2-(9H-fluoren-9-ylmethoxycarbonylamino)propanoic acid
**Y1**	170642-27-0	(2R)-2-(9H-fluoren-9-ylmethoxycarbonylamino)butanoic acid
**Y2**	146549-21-5	(2S)-2-(9H-fluoren-9-ylmethoxycarbonylamino)pent-4-enoic acid
**Y3**	170642-28-1	(2R)-2-(9H-fluoren-9-ylmethoxycarbonylamino)pent-4-enoic acid
**Y4**	144701-24-6	(2R)-2-(9H-fluoren-9-ylmethoxycarbonylamino)pentanoic acid
**Y5**	84624-17-9	(2R)-2-(9H-fluoren-9-ylmethoxycarbonylamino)-3-methylbutanoic acid
**Y6**	71989-23-6	(2S,3S)-2-(9H-fluoren-9-ylmethoxycarbonylamino)-3-methylpentanoic acid
**Y7**	204320-60-5	(2R)-2-(9H-fluoren-9-ylmethoxycarbonylamino)-5-methylhexanoic acid
**Y8**	180414-94-2	(2S)-2-(9H-fluoren-9-ylmethoxycarbonylamino)-5-methylhexanoic acid
**Y9**	139551-74-9	(2S)-2-(9H-fluoren-9-ylmethoxycarbonylamino)-4,4-dimethylpentanoic acid
**Y10**	1212257-18-5	(2S)-2-cyclopropyl-2-(9H-fluoren-9-ylmethoxycarbonylamino)acetic acid
**Y11**	220497-61-0	(2S)-2-cyclopentyl-2-(9H-fluoren-9-ylmethoxycarbonylamino)acetic acid

**Y12**	198543-96-3	(2R)-2-cyclohexyl-2-(9H-fluoren-9-ylmethoxycarbonylamino)acetic acid
**Y13**	135673-97-1	(2S)-3-cyclohexyl-2-(9H-fluoren-9-ylmethoxycarbonylamino)propanoic acid
**Y14**	35737-10-1	3-(9H-fluoren-9-ylmethoxycarbonylamino)propanoic acid
**Y15**	77128-70-2	2-[9H-fluoren-9-ylmethoxycarbonyl(methyl)amino]acetic acid
**Y16**	193954-26-6	(3S)-3-(9H-fluoren-9-ylmethoxycarbonylamino)butanoic acid
**Y17**	88574-06-5	6-(9H-fluoren-9-ylmethoxycarbonylamino)hexanoic acid
**Y18**	234442-58-1	2-[butyl(9H-fluoren-9-ylmethoxycarbonyl)amino]acetic acid
**Y19**	103478-62-2	(2S)-2-[9H-fluoren-9-ylmethoxycarbonyl(methyl)amino]-4-methylpentanoic acid
**Y20**	193693-64-0	1-(9H-fluoren-9-ylmethoxycarbonyl)azetidine-3-carboxylic acid
**Y21**	71989-31-6	(2S)-1-(9H-fluoren-9-ylmethoxycarbonyl)pyrrolidine-2-carboxylic acid
**Y22**	193693-68-4	(3S)-1-(9H-fluoren-9-ylmethoxycarbonyl)piperidine-3-carboxylic acid
**Y23**	180181-05-9	2-[1-(9H-fluoren-9-ylmethoxycarbonyl)piperidin-4-yl]acetic acid
**Y24**	180576-05-0	2-[4-(9H-fluoren-9-ylmethoxycarbonyl)piperazin-1-yl]acetic acid
**Y25**	71989-33-8	(2S)-2-(9H-fluoren-9-ylmethoxycarbonylamino)-3-[(2-methylpropan-2-yl)oxy]propanoic acid
**Y26**	159766-56-0	(2S)-6-acetamido-2-(9H-fluoren-9-ylmethoxycarbonylamino)hexanoic acid
**Y27**	111061-55-3	(2S)-2-(9H-fluoren-9-ylmethoxycarbonylamino)-4-trityloxybutanoic acid
**Y28**	180570-71-2	(2R)-2-(9H-fluoren-9-ylmethoxycarbonylamino)-4-oxo-4-(tritylamino)butanoic acid
**Y29**	197632-77-2	(2S)-2-[9H-fluoren-9-ylmethoxycarbonyl(methyl)amino]-3-[(2-methylpropan-2-yl)oxy]propanoic acid
**Y30**	71989-28-1	(2S)-2-(9H-fluoren-9-ylmethoxycarbonylamino)-4-methylsulfanylbutanoic acid
**Y31**	102410-65-1	(2S)-2-(9H-fluoren-9-ylmethoxycarbonylamino)-2-phenylacetic acid
**Y32**	211637-74-0	(2S)-2-(9H-fluoren-9-ylmethoxycarbonylamino)-3-(3-methylphenyl)propanoic acid
**Y33**	132684-59-4	(2S)-2-(9H-fluoren-9-ylmethoxycarbonylamino)-4-phenylbutanoic acid
**Y34**	112883-43-9	(2S)-2-(9H-fluoren-9-ylmethoxycarbonylamino)-3-naphthalen-2-ylpropanoic acid
**Y35**	138774-94-4	(2R)-2-(9H-fluoren-9-ylmethoxycarbonylamino)-3-naphthalen-2-ylpropanoic acid

**Y36**	199110-64-0	(2S)-2-(9H-fluoren-9-ylmethoxycarbonylamino)-3-(4-phenylphenyl)propanoic acid
**Y37**	201484-50-6	(2S)-2-(9H-fluoren-9-ylmethoxycarbonylamino)-3,3-diphenylpropanoic acid
**Y38**	185116-42-1	3-(9H-fluoren-9-ylmethoxycarbonylamino)benzoic acid
**Y39**	185116-43-2	4-(9H-fluoren-9-ylmethoxycarbonylamino)benzoic acid
**Y40**	164470-64-8	4-[(9H-fluoren-9-ylmethoxycarbonylamino)methyl]benzoic acid
**Y41**	176504-01-1	2-[4-[(9H-fluoren-9-ylmethoxycarbonylamino)methyl]phenyl]acetic acid
**Y42**	198560-38-2	(2S)-1-(9H-fluoren-9-ylmethoxycarbonyl)-2,3-dihydroindole-2-carboxylic acid
**Y43**	136030-33-6	(3S)-2-(9H-fluoren-9-ylmethoxycarbonyl)-3,4-dihydro-1H-isoquinoline-3-carboxylic acid
**Y44**	185379-40-2	(2S)-2-(9H-fluoren-9-ylmethoxycarbonylamino)-3-pyridin-2-ylpropanoic acid
**Y45**	81379-52-4	(2S)-2-(9H-fluoren-9-ylmethoxycarbonylamino)-3-[1-[(2-methylpropan-2-yl)oxycarbonyl]imidazol-4-yl]propanoic acid
**Y46**	135610-90-1	(2R)-2-(9H-fluoren-9-ylmethoxycarbonylamino)-3-(1-tritylimidazol-4-yl)propanoic acid
**Y47**	268731-07-3	(3R)-2-(9H-fluoren-9-ylmethoxycarbonyl)-1,3,4,9-tetrahydropyrido[3,4-b]indole-3-carboxylic acid
**Y48**	177966-63-1	(2R)-2-(9H-fluoren-9-ylmethoxycarbonylamino)-3-(4-nitrophenyl)propanoic acid
**Y49**	95753-55-2	(2S)-2-(9H-fluoren-9-ylmethoxycarbonylamino)-3-(4-nitrophenyl)propanoic acid
**Y50**	117666-96-3	(2S)-3-(4-benzoylphenyl)-2-(9H-fluoren-9-ylmethoxycarbonylamino)propanoic acid
**Y51**	86060-83-5	(3S)-3-(9H-fluoren-9-ylmethoxycarbonylamino)-4-oxo-4-phenylmethoxybutanoic acid
**Y52**	212783-75-0	2-[[2-(9H-fluoren-9-ylmethoxycarbonylamino)-6-tricyclo[9.4.0.03,8]pentadeca-1(15),3(8),4,6,11,13-hexaenyl]oxy]acetic acid
**Y53**	130309-35-2	(2S)-2-(9H-fluoren-9-ylmethoxycarbonylamino)-3-thiophen-2-ylpropanoic acid
**Y54**	177966-60-8	(2S)-3-(1-benzothiophen-3-yl)-2-(9H-fluoren-9-ylmethoxycarbonylamino)propanoic acid
**Y55**	198560-43-9	(2S)-3-(3,4-difluorophenyl)-2-(9H-fluoren-9-ylmethoxycarbonylamino)propanoic acid
**Y56**	352523-16-1	(2S)-2-(9H-fluoren-9-ylmethoxycarbonylamino)-3-[2-(trifluoromethyl)phenyl]propanoic acid
**Y57**	247113-86-6	(2S)-2-(9H-fluoren-9-ylmethoxycarbonylamino)-3-[4-(trifluoromethyl)phenyl]propanoic acid
**Y58**	352351-62-3	(2S)-3-(2,4-dichlorophenyl)-2-(9H-fluoren-9-ylmethoxycarbonylamino)propanoic acid
**Y59**	177966-58-4	(2R)-3-(3,4-dichlorophenyl)-2-(9H-fluoren-9-ylmethoxycarbonylamino)propanoic acid

**Z1**	29022-11-5	2-(9H-fluoren-9-ylmethoxycarbonylamino)acetic acid
**Z2**	35661-39-3	(2S)-2-(9H-fluoren-9-ylmethoxycarbonylamino)propanoic acid
**Z3**	79990-15-1	(2R)-2-(9H-fluoren-9-ylmethoxycarbonylamino)propanoic acid
**Z4**	68858-20-8	(2S)-2-(9H-fluoren-9-ylmethoxycarbonylamino)-3-methylbutanoic acid
**Z5**	112883-41-7	(2R)-2-(9H-fluoren-9-ylmethoxycarbonylamino)hexanoic acid
**Z6**	198543-64-5	(2R)-2-(9H-fluoren-9-ylmethoxycarbonylamino)-3,3-dimethylbutanoic acid
**Z7**	35661-60-0	(2S)-2-(9H-fluoren-9-ylmethoxycarbonylamino)-4-methylpentanoic acid
**Z8**	77284-32-3	(2S)-2-(9H-fluoren-9-ylmethoxycarbonylamino)hexanoic acid
**Z9**	132684-60-7	(2S)-2-(9H-fluoren-9-ylmethoxycarbonylamino)-3,3-dimethylbutanoic acid
**Z10**	144701-25-7	(2R)-3-cyclohexyl-2-(9H-fluoren-9-ylmethoxycarbonylamino)propanoic acid
**Z11**	269078-73-1	(2S)-4-cyclohexyl-2-(9H-fluoren-9-ylmethoxycarbonylamino)butanoic acid
**Z12**	94744-50-0	2-(9H-fluoren-9-ylmethoxycarbonylamino)-2-methylpropanoic acid
**Z13**	84000-07-7	(2S)-2-[9H-fluoren-9-ylmethoxycarbonyl(methyl)amino]propanoic acid
**Z14**	172965-84-3	3-[9H-fluoren-9-ylmethoxycarbonyl(methyl)amino]propanoic acid
**Z15**	84000-11-3	(2S)-2-[9H-fluoren-9-ylmethoxycarbonyl(methyl)amino]-3-methylbutanoic acid
**Z16**	885951-77-9	1-(9H-fluoren-9-ylmethoxycarbonylamino)cyclobutane-1-carboxylic acid
**Z17**	1263045-62-0	1-[(9H-fluoren-9-ylmethoxycarbonylamino)methyl]cyclopropane-1-carboxylic acid
**Z18**	101555-62-8	(2R)-1-(9H-fluoren-9-ylmethoxycarbonyl)pyrrolidine-2-carboxylic acid
**Z19**	71989-31-6	(2S)-1-(9H-fluoren-9-ylmethoxycarbonyl)pyrrolidine-2-carboxylic acid
**Z20**	101555-63-9	(2R)-1-(9H-fluoren-9-ylmethoxycarbonyl)piperidine-2-carboxylic acid
**Z21**	188715-40-4	4-[(9H-fluoren-9-ylmethoxycarbonylamino)methyl]cyclohexane-1-carboxylic acid
**Z22**	130309-37-4	(2S,3aS,7aS)-1-(9H-fluoren-9-ylmethoxycarbonyl)-2,3,3a,4,5,6,7,7a-octahydroindole-2-carboxylic acid
**Z23**	91000-69-0	(2S)-5-(diaminomethylideneamino)-2-(9H-fluoren-9-ylmethoxycarbonylamino)pentanoic acid
**Z24**	71989-35-0	(2S,3R)-2-(9H-fluoren-9-ylmethoxycarbonylamino)-3-[(2-methylpropan-2-yl)oxy]butanoic acid

**Z25**	132388-59-1	(2S)-2-(9H-fluoren-9-ylmethoxycarbonylamino)-4-oxo-4-(tritylamino)butanoic acid
**Z26**	200623-62-7	(2R)-2-(9H-fluoren-9-ylmethoxycarbonylamino)-5-oxo-5-(tritylamino)pentanoic acid
**Z27**	132327-80-1	(2S)-2-(9H-fluoren-9-ylmethoxycarbonylamino)-5-oxo-5-(tritylamino)pentanoic acid
**Z28**	312965-04-1	4-(9H-fluoren-9-ylmethoxycarbonyl)morpholine-2-carboxylic acid
**Z29**	285996-72-7	4-(9H-fluoren-9-ylmethoxycarbonylamino)oxane-4-carboxylic acid
**Z30**	76265-70-8	(2S)-2-(9H-fluoren-9-ylmethoxycarbonylamino)-4-methylsulfinylbutanoic acid
**Z31**	198545-89-0	(4S)-3-(9H-fluoren-9-ylmethoxycarbonyl)-1,3-thiazolidine-4-carboxylic acid
**Z32**	86123-10-6	(2R)-2-(9H-fluoren-9-ylmethoxycarbonylamino)-3-phenylpropanoic acid
**Z33**	135944-09-1	(2R)-2-(9H-fluoren-9-ylmethoxycarbonylamino)-4-phenylbutanoic acid
**Z34**	159610-82-9	(E,2S)-2-(9H-fluoren-9-ylmethoxycarbonylamino)-5-phenylpent-4-enoic acid
**Z35**	205526-38-1	(2R)-2-(9H-fluoren-9-ylmethoxycarbonylamino)-3-(4-phenylphenyl)propanoic acid
**Z36**	150256-42-1	2-(9H-fluoren-9-ylmethoxycarbonylamino)benzoic acid
**Z37**	141743-13-7	2-[benzyl(9H-fluoren-9-ylmethoxycarbonyl)amino]acetic acid
**Z38**	138775-05-0	(2R)-2-[9H-fluoren-9-ylmethoxycarbonyl(methyl)amino]-3-phenylpropanoic acid
**Z39**	77128-73-5	(2S)-2-[9H-fluoren-9-ylmethoxycarbonyl(methyl)amino]-3-phenylpropanoic acid
**Z40**	130309-33-0	(3R)-2-(9H-fluoren-9-ylmethoxycarbonyl)-3,4-dihydro-1H-isoquinoline-3-carboxylic acid
**Z41**	372159-75-6	3-(9H-fluoren-9-ylmethoxycarbonylamino)naphthalene-2-carboxylic acid
**Z42**	143824-78-6	(2S)-2-(9H-fluoren-9-ylmethoxycarbonylamino)-3-[1-[(2-methylpropan-2-yl)oxycarbonyl]indol-3-yl]propanoic acid
**Z43**	197632-75-0	(2S)-2-[9H-fluoren-9-ylmethoxycarbonyl(methyl)amino]-3-[1-[(2-methylpropan-2-yl)oxycarbonyl]indol-3-yl]propanoic acid
**Z44**	159611-02-6	(2S)-2-(9H-fluoren-9-ylmethoxycarbonylamino)-3-(furan-2-yl)propanoic acid
**Z45**	77128-72-4	(2S)-2-(9H-fluoren-9-ylmethoxycarbonylamino)-3-(4-methoxyphenyl)propanoic acid
**Z46**	122889-11-6	(2R)-2-(9H-fluoren-9-ylmethoxycarbonylamino)-3-phenylmethoxypropanoic acid
**Z47**	118488-18-9	(2R)-2-(9H-fluoren-9-ylmethoxycarbonylamino)-3-[4-[(2-methylpropan-2-yl)oxy]phenyl]propanoic acid
**Z48**	71989-38-3	(2S)-2-(9H-fluoren-9-ylmethoxycarbonylamino)-3-[4-[(2-methylpropan-2-yl)oxy]phenyl]propanoic acid

**Z49**	71989-40-7	(2S)-2-(9H-fluoren-9-ylmethoxycarbonylamino)-3-(4-phenylmethoxyphenyl)propanoic acid
**Z50**	174800-02-3	(2S,4R)-1-(9H-fluoren-9-ylmethoxycarbonyl)-4-phenylmethoxypyrrolidine-2-carboxylic acid
**Z51**	205526-26-7	(2S)-2-(9H-fluoren-9-ylmethoxycarbonylamino)-3-(2-fluorophenyl)propanoic acid
**Z52**	198560-68-8	(2S)-2-(9H-fluoren-9-ylmethoxycarbonylamino)-3-(3-fluorophenyl)propanoic acid
**Z53**	479064-89-6	(3S)-3-(9H-fluoren-9-ylmethoxycarbonylamino)-3-(4-fluorophenyl)propanoic acid
**Z54**	198560-44-0	(2S)-3-(3-chlorophenyl)-2-(9H-fluoren-9-ylmethoxycarbonylamino)propanoic acid
**Z55**	205526-24-5	(2S)-3-(3,5-difluorophenyl)-2-(9H-fluoren-9-ylmethoxycarbonylamino)propanoic acid
**Z56**	198561-04-5	(2S)-3-(4-bromophenyl)-2-(9H-fluoren-9-ylmethoxycarbonylamino)propanoic acid
**Z57**	82565-68-2	(2S)-2-(9H-fluoren-9-ylmethoxycarbonylamino)-3-(4-iodophenyl)propanoic acid
**Z58**	96402-49-2	(2S)-2-(9H-fluoren-9-ylmethoxycarbonylamino)-3-naphthalen-1-ylpropanoic acid
**Z59**	204322-23-6	(3S)-2-(9H-fluoren-9-ylmethoxycarbonyl)-1,3,4,9-tetrahydropyrido[3,4-b]indole-3-carboxylic acid
**CA1**	503-74-2	3-methylbutanoic acid
**CA2**	98-89-5	cyclohexanecarboxylic acid
**CA3**	1118-68-9	2-(dimethylamino)acetic acid
**CA4**	6914-79-0	1-cyanocyclopropane-1-carboxylic acid
**CA5**	5337-03-1	oxane-4-carboxylic acid
**CA6**	68947-43-3	1-methylpiperidine-4-carboxylic acid
**CA7**	79-14-1	2-hydroxyacetic acid
**CA8**	627-03-2	2-ethoxyacetic acid
**CA9**	16874-33-2	oxolane-2-carboxylic acid
**CA10**	61826-55-9	cis-3-acetyl-2,2-dimethylcyclobutaneacetic acid
**CA11**	280772-00-1	1-methylsulfonylpiperidine-4-carboxylic acid
**CA12**	406-93-9	4,4,4-trifluorobutanoic acid
**CA13**	6120-95-2	1-phenylcyclopropane-1-carboxylic acid

**CA14**	939-90-2	trans-2-phenyl-1-cyclopropanecarboxylic acid
**CA15**	634-97-9	1H-pyrrole-2-carboxylic acid
**CA16**	1621-91-6	1H-pyrazole-5-carboxylic acid
**CA17**	98-97-5	pyrazine-2-carboxylic acid
**CA18**	22884-10-2	2-imidazol-1-ylacetic acid
**CA19**	99-96-7	4-hydroxybenzoic acid
**CA20**	30379-55-6	2-phenylmethoxyacetic acid
**CA21**	1670-83-3	1H-indole-7-carboxylic acid
**CA22**	2124-55-2	1H-indole-4-carboxylic acid
**CA23**	1477-50-5	1H-indole-2-carboxylic acid
**CA24**	139022-25-6	imidazo[1,2-a]pyridine-6-carboxylic acid
**CA25**	156270-06-3	1H-pyrrolo[2,3-b]pyridine-3-carboxylic acid
**CA26**	879-65-2	quinoxaline-2-carboxylic acid
**CA27**	24297-59-4	2-indol-1-ylacetic acid
**CA28**	87-51-4	2-(1H-indol-3-yl)acetic acid
**CA29**	50890-83-0	1-methylindazole-3-carboxylic acid
**CA30**	16209-00-0	4-pyrazol-1-ylbenzoic acid
**CA31**	956317-36-5	5-methyl-2-(triazol-2-yl)benzoic acid
**CA32**	22300-56-7	5-methyl-2-phenyltriazole-4-carboxylic acid
**CA33**	354813-15-3	4-(4-methylpiperazin-1-yl)benzoic acid
**CA34**	88-14-2	furan-2-carboxylic acid
**CA35**	3405-77-4	5-methyl-1,2-oxazole-3-carboxylic acid
**CA36**	6338-41-6	5-(hydroxymethyl)furan-2-carboxylic acid
**CA37**	122-59-8	2-phenoxyacetic acid

**CA38**	17199-29-0	(2S)-2-hydroxy-2-phenylacetic acid
**CA39**	100-09-4	4-methoxybenzoic acid
**CA40**	38116-61-9	6-methyl-2-oxo-1H-pyridine-3-carboxylic acid
**CA41**	556-08-1	4-acetamidobenzoic acid
**CA42**	93-40-3	2-(3,4-dimethoxyphenyl)acetic acid
**CA43**	496-41-3	1-benzofuran-2-carboxylic acid
**CA44**	4865-84-3	2-(1,2-benzoxazol-3-yl)acetic acid
**CA45**	2861-28-1	2-(1,3-benzodioxol-5-yl)acetic acid
**CA46**	14441-90-8	5-phenyl-1,2-oxazole-3-carboxylic acid
**CA47**	42106-48-9	2-morpholin-4-ylbenzoic acid
**CA48**	215309-00-5	3-morpholin-4-ylbenzoic acid
**CA49**	3739-38-6	3-phenoxybenzoic acid
**CA50**	62642-62-0	4-(morpholin-4-ylmethyl)benzoic acid
**CA51**	3622-35-3	1,3-benzothiazole-6-carboxylic acid
**CA52**	74-11-3	4-chlorobenzoic acid
**CA53**	455-40-3	3,5-difluorobenzoic acid
**CA54**	24065-33-6	5-chlorothiophene-2-carboxylic acid
**CA55**	1878-66-6	2-(4-chlorophenyl)acetic acid
**CA56**	131747-42-7	6-(trifluoromethyl)pyridine-2-carboxylic acid
**CA57**	399-76-8	5-fluoro-1H-indole-2-carboxylic acid
**CA58**	10517-21-2	5-chloro-1H-indole-2-carboxylic acid
**CA59**	182181-19-7	6-chloroimidazo[1,2-a]pyridine-2-carboxylic acid

Table S4. Summary of LC-MS analysis of randomly selected monomers from the linear thiol-peptide library. Peptides were analyzed using a gradient 10–60% ACN over 5 min. Gradient start time at 1 min, gradient end time at 6 min, MS detection between 1.5–6.1 min.

plate and well of peptide synthesis	monomer ID in Library 1	monomer ID in Library 2	$[M+H]^+$ calculated	found	retention time [min]	comment
**1_B5**	M745	M29	593.2174			not found
**1_D10**	M915	M82	615.2453	615.40	2.820	
**1_F12**	M337	M132	715.2906	715.45	4.580	
**1_G20**	M577	M164	712.2961	712.75	3.240	
**1_H20**	M339	M188	645.2851	645.40	4.160	
**1_J8**	M1061	M224	654.3317	654.45	2.727	
**1_L10**	M219	M274	712.3040	712.15	3.260	
**1_N18**	M341	M330	584.2146	584.25	4.147	
**1_O14**	M53	M350	696.259	696.45	3.913	
**1_P22**	M1327	M382	633.2739	633.20	4.980	
**2_A4**	M847	M388	610.3055	610.45	4.173	
**2_A19**	M440	M403	652.2949			not found
**2_C17**	M1137	M449	739.3077			possible product peak outside ms detection time
**2_C21**	M897	M453	594.2742	594.35	5.227	
**2_D2**	M1232	M458	624.2039	624.15	4.333	
**2_D9**	M1163	M465	796.3372	796.25	4.547	
**2_D24**	M630	M480	528.2848	528.35	2.847	
**2_E14**	M1278	M494	656.2124			not found
**2_F16**	M631	M520	593.3001	593.40	3.913	
**2_G7**	M1190	M535	565.2721	565.35	3.173	

**2_H6**	M1448	M558	732.2960			not found
**2_H14**	M512	M566	692.3262	692.20	5.713	
**2_H18**	M1307	M570	711.3572			not found
**2_I2**	M514	M578	558.2200	558.25	4.047	
**2_I6**	M561	M582	582.249	582.35	3.500	
**2_N19**	M517	M715	688.3524	688.55	3.460	
**2_O12**	M973	M732	609.3215	609.30	4.200	
**2_O18**	M732	M738	603.3320	603.60	3.707	
**2_P3**	M134	M747	622.2725			not found
**2_P6**	M1191	M750	652.2597	652.20	4.007	
**3_B5**	M494	M797	761.3113	761.25	4.767	
**3_B8**	M1501	M800	716.3541	716.50	5.600	
**3_B19**	M520	M811	603.2514	603.20	3.107	main m/z species in product peak not desired peptide
**3_B24**	M323	M816	658.3994	658.30	5.253	
**3_C12**	M807	M828	696.3023	696.20	6.100	
**3_C16**	M1025	M832	652.3273	652.25	3.033	
**3_C18**	M398	M834	723.4259	723.60	4.053	main m/z species in product peak not desired peptide
**3_F9**	M563	M897	842.2901			possible product peak outside ms detection time
**3_G8**	M683	M920	786.2884	786.20	4.947	
**3_H23**	M1193	M959	539.3007			not found
**3_I6**	M756	M966	808.3372			possible product peak outside ms detection time
**3_I9**	M685	M969	546.1934	546.30	4.433	
**3_J3**	M474	M987	891.1734			not found
**3_J14**	M207	M998	602.3004	602.40	3.940	

**3_J17**	M1338	M1001	491.2684	491.30	3.993	
**3_K16**	M567	M1024	682.2822			possible product peak outside ms detection time
**3_K23**	M329	M1031	730.2382	730.15	4.513	
**3_M3**	M523	M1059	764.3837			not found
**3_M19**	M1047	M1075	668.3474			not found
**3_P24**	M332	M1152	779.1751			possible product peak outside ms detection time
**4_A10**	M1147	M1162	607.1710	607.25	4.113	main m/z species in product peak not desired peptide
**4_B12**	M547	M1188	742.1636	742.35	5.840	
**4_D16**	M667	M1240	553.2225	553.35	2.560	
**4_D22**	M617	M1246	581.2426	581.40	4.100	
**4_E17**	M711	M1265	599.3623	599.50	4.967	
**4_E20**	M1459	M1268	805.1889			possible product peak outside ms detection time
**4_F16**	M743	M1288	749.2572			not found
**4_F19**	M118	M1291	739.3885	739.30	4.860	
**4_F24**	M1291	M1296	666.3793	666.50	2.520	
**4_I21**	M286	M1365	696.3211	696.45	5.300	
**4_J6**	M213	M1374	757.2834	757.25	5.047	
**4_J9**	M715	M1377	544.2222	544.30	2.880	
**4_J23**	M1485	M1391	707.2819	707.50	4.420	
**4_L1**	M19	M1417	746.1996			not found
**4_N7**	M358	M1471	648.2484	648.45	3.420	
**4_N12**	M1126	M1476	699.2957	699.45	4.127	
**4_N16**	M551	M1480	719.3219	719.25	5.447	
**4_O9**	M744	M1497	756.3109			possible product peak outside ms detection time

**4_O18**	M1080	M1506	709.2025	709.15	4.693		
**4_P8**	M1008	M1520	813.2343				not found

SUPPLEMENTARY FIGURES

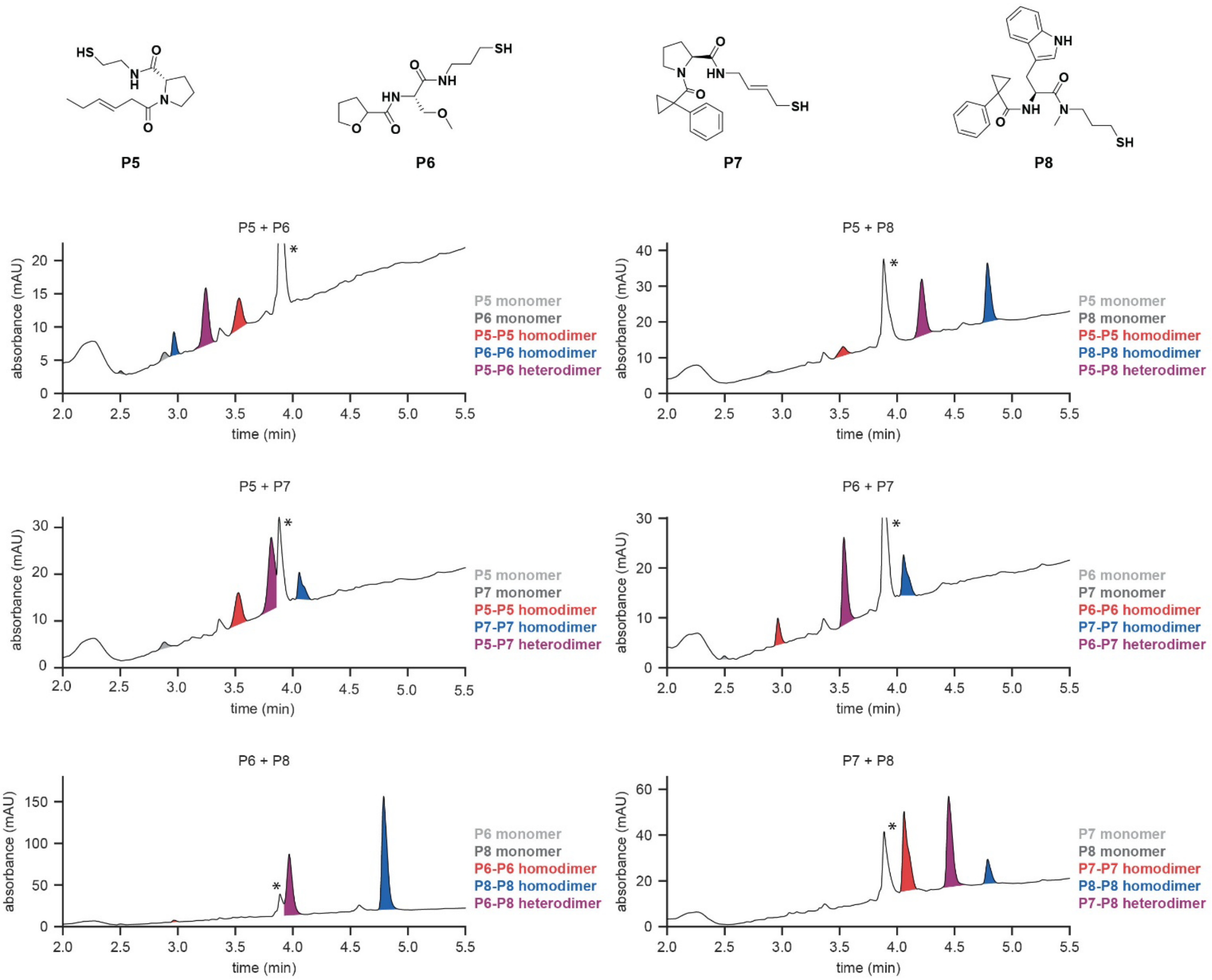

Figure S1. LC-MS chromatogram traces of test reactions with crude peptides P5–P8. DMSO stock of peptides were combined and incubated for 24 h in 60 mM NH_4HCO_3 buffer containing 50% DMSO. The final monomer concentration was 100 μM each. Asterisk indicates a contamination compound of the column. Gradient used for separation 0–60% ACN.

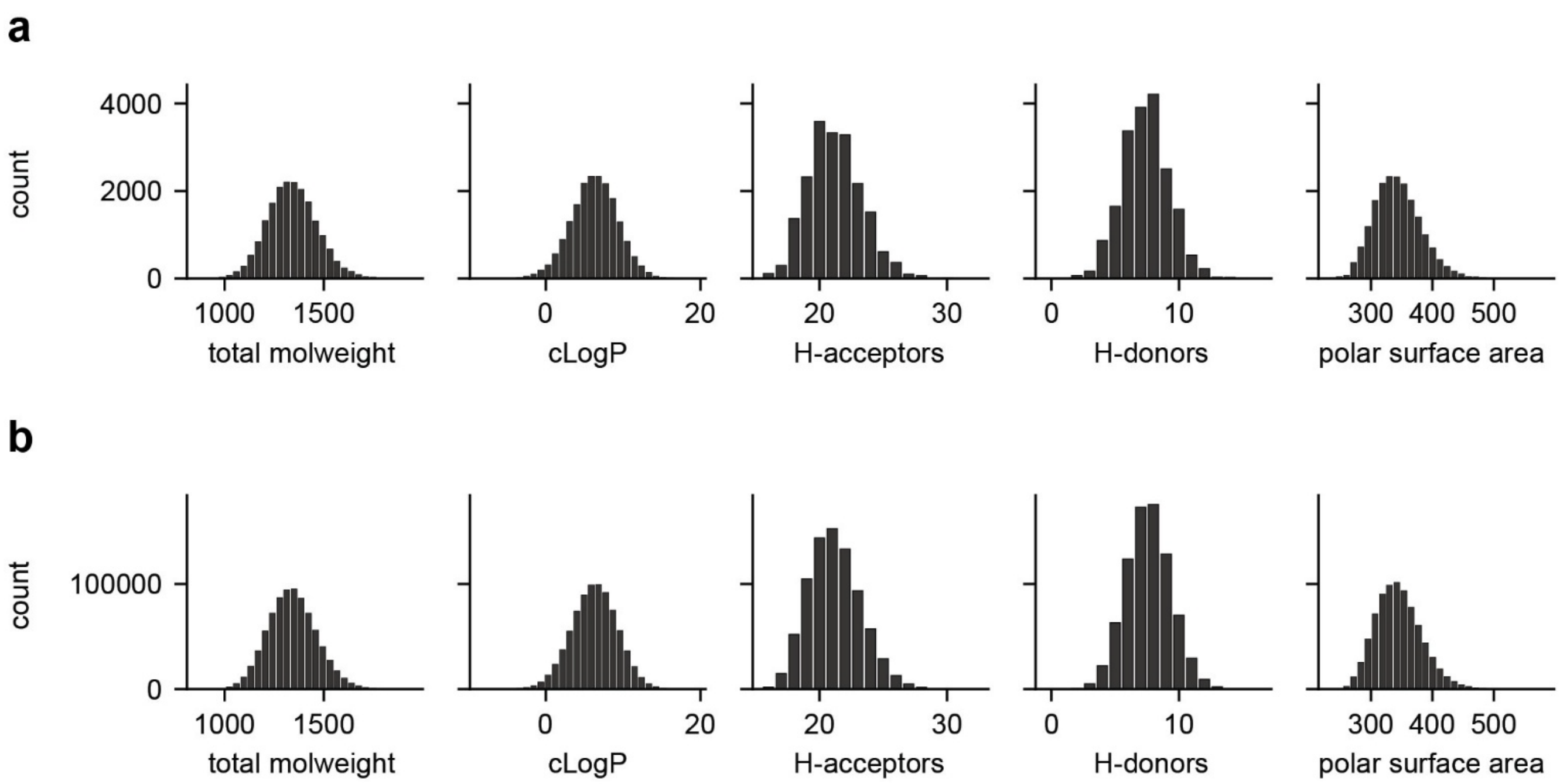

Figure S2. Properties of screened peptide pairs. Properties were calculated using DataWarrior. a) Peptide pairs of Library 1. b) Peptide pairs of Library 2.

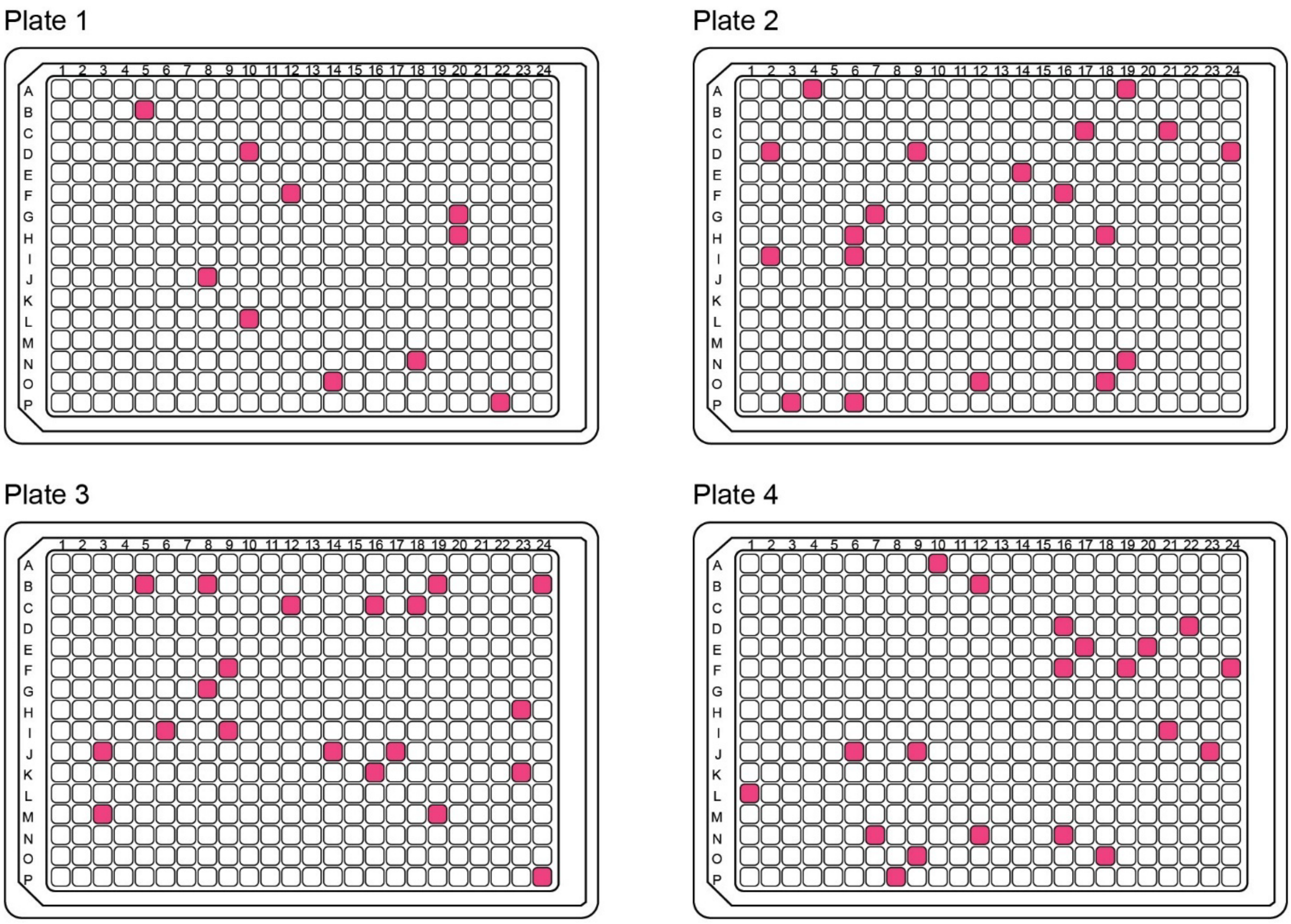

Figure S3. Random selection of thiol-containing peptides for quality analysis by LC-MS.

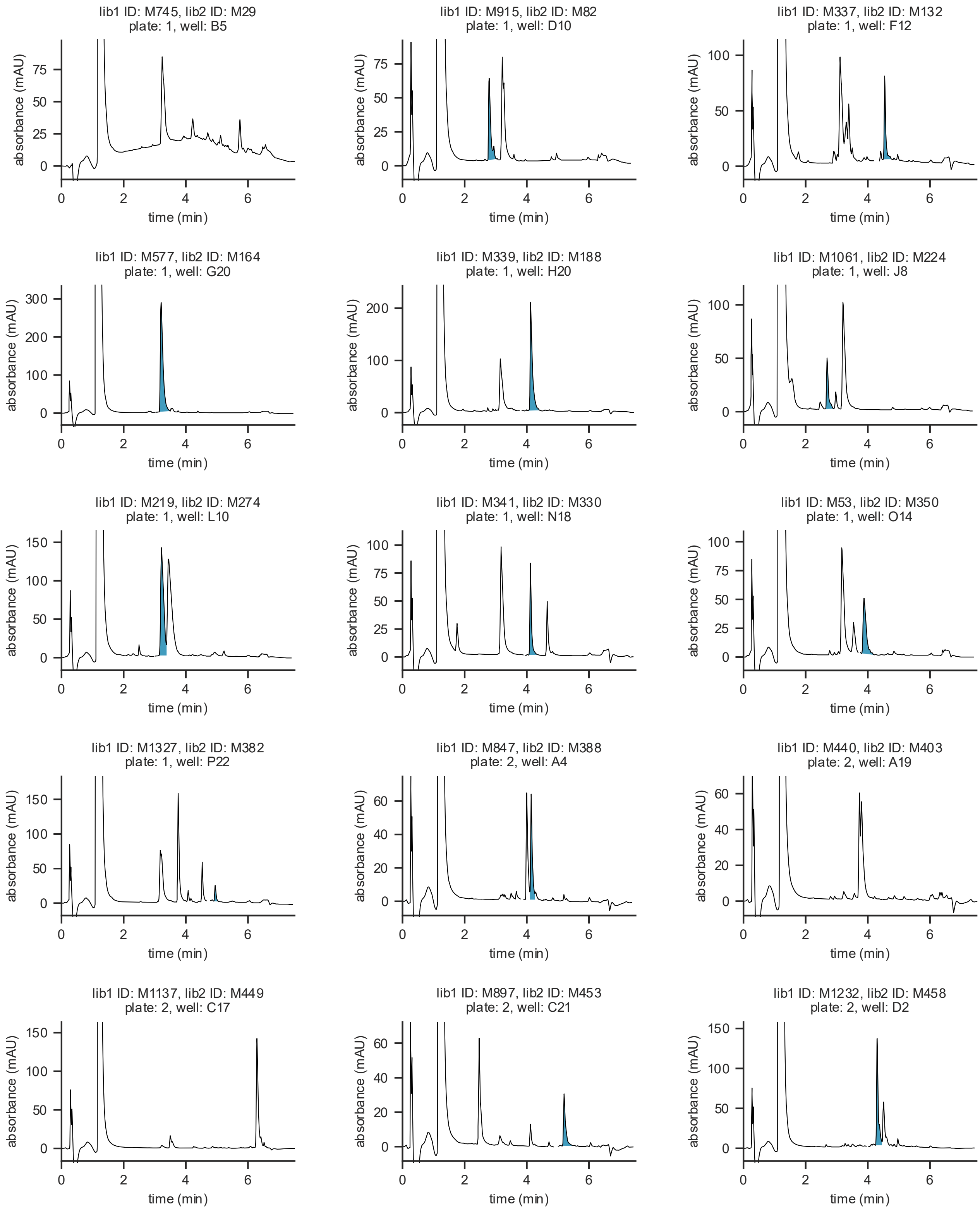

Figure S4. Quality analysis of synthesized thiol peptide library. LC-MS analysis of randomly selected monomers from the thiol-functionalized linear peptide library. Peptides were analyzed using a gradient of 10–60% ACN over 5 min. Gradient start time at 1 min, gradient end time at 6 min, MS detection between 1.5–6.1 min. The peak of the desired product peak is highlighted in blue. Some peptides were analyzed before BDT removal (peak at 3.5 min elution time) and some after BDT removal.

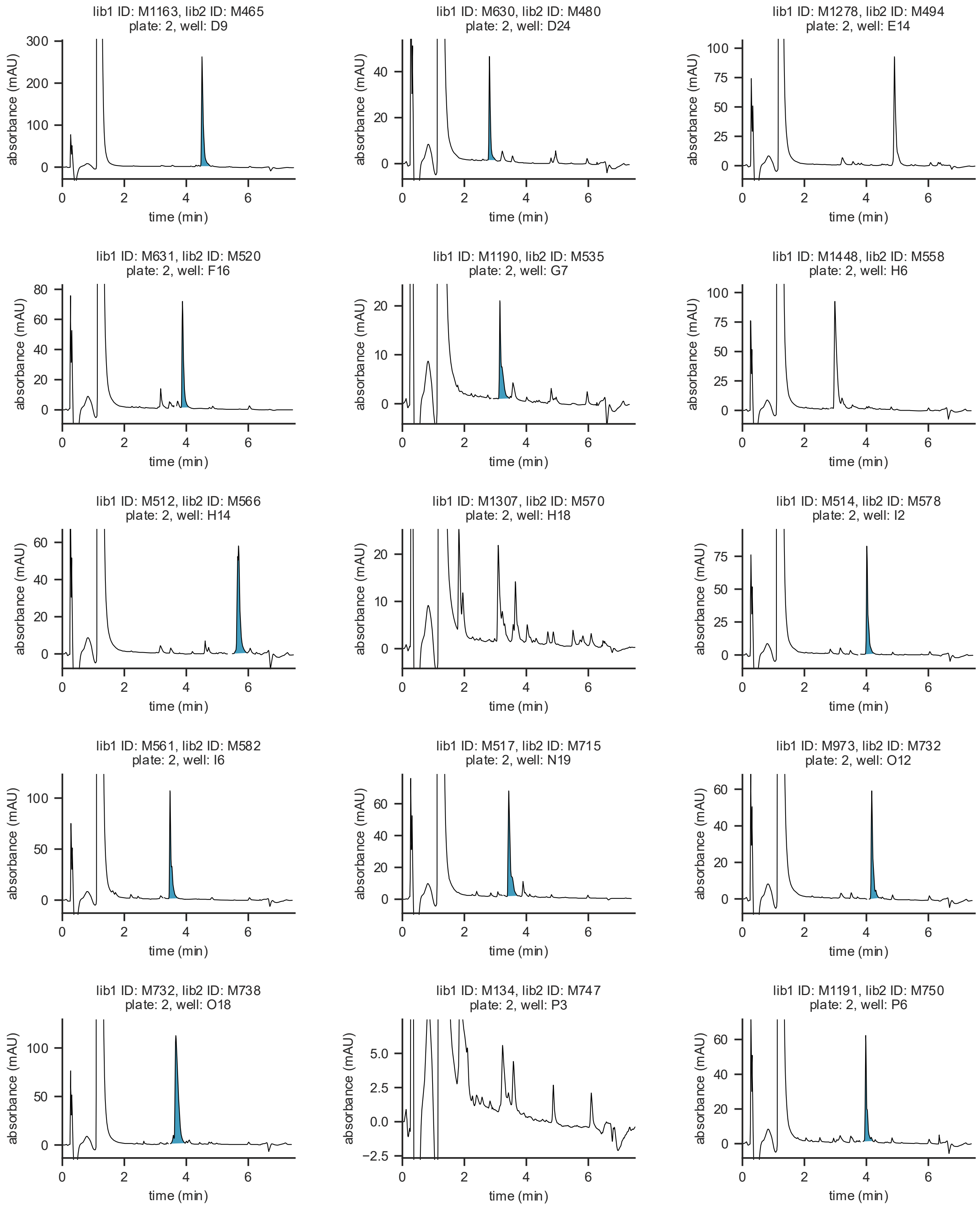

Figure S4. Continued

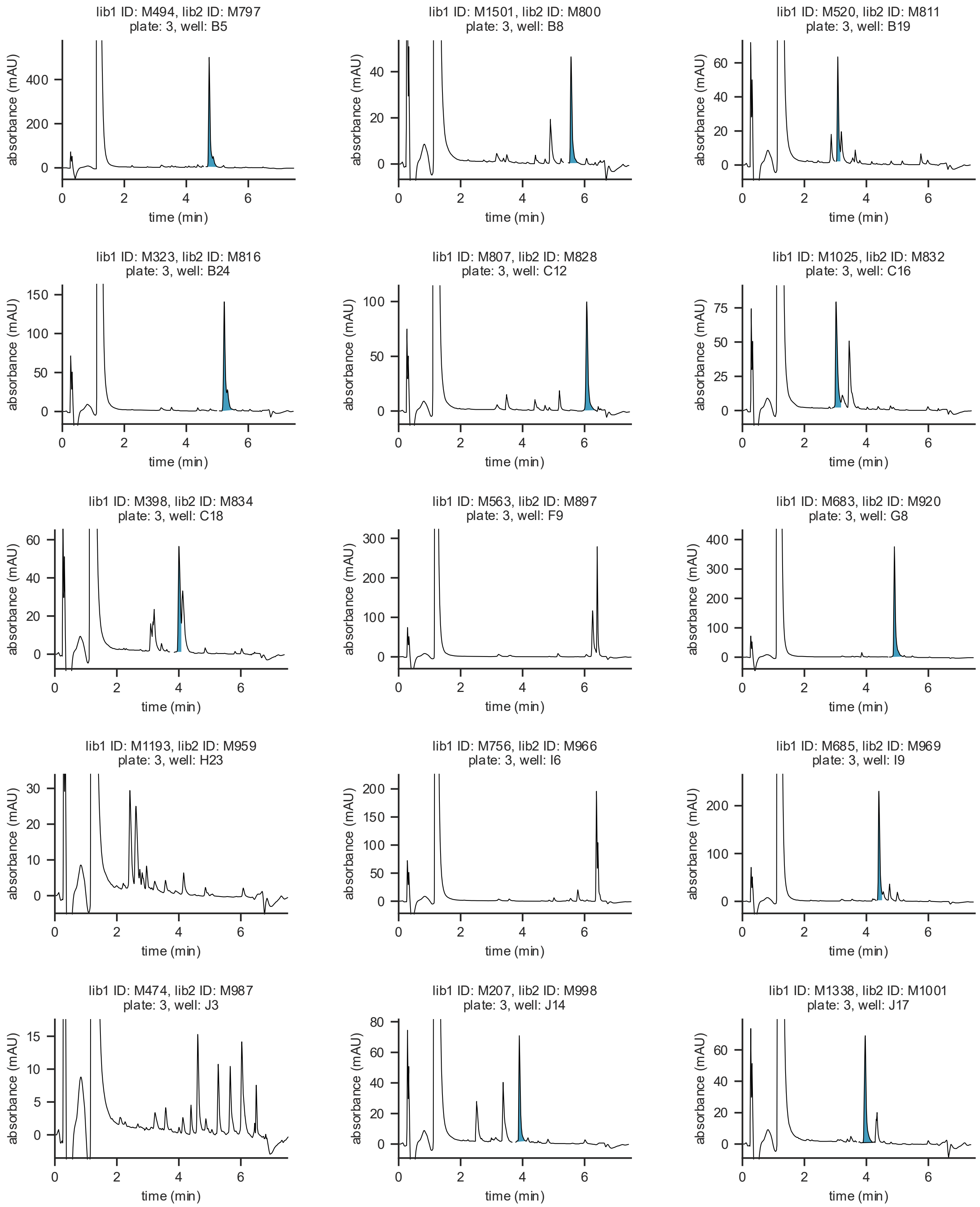

Figure S4. Continued

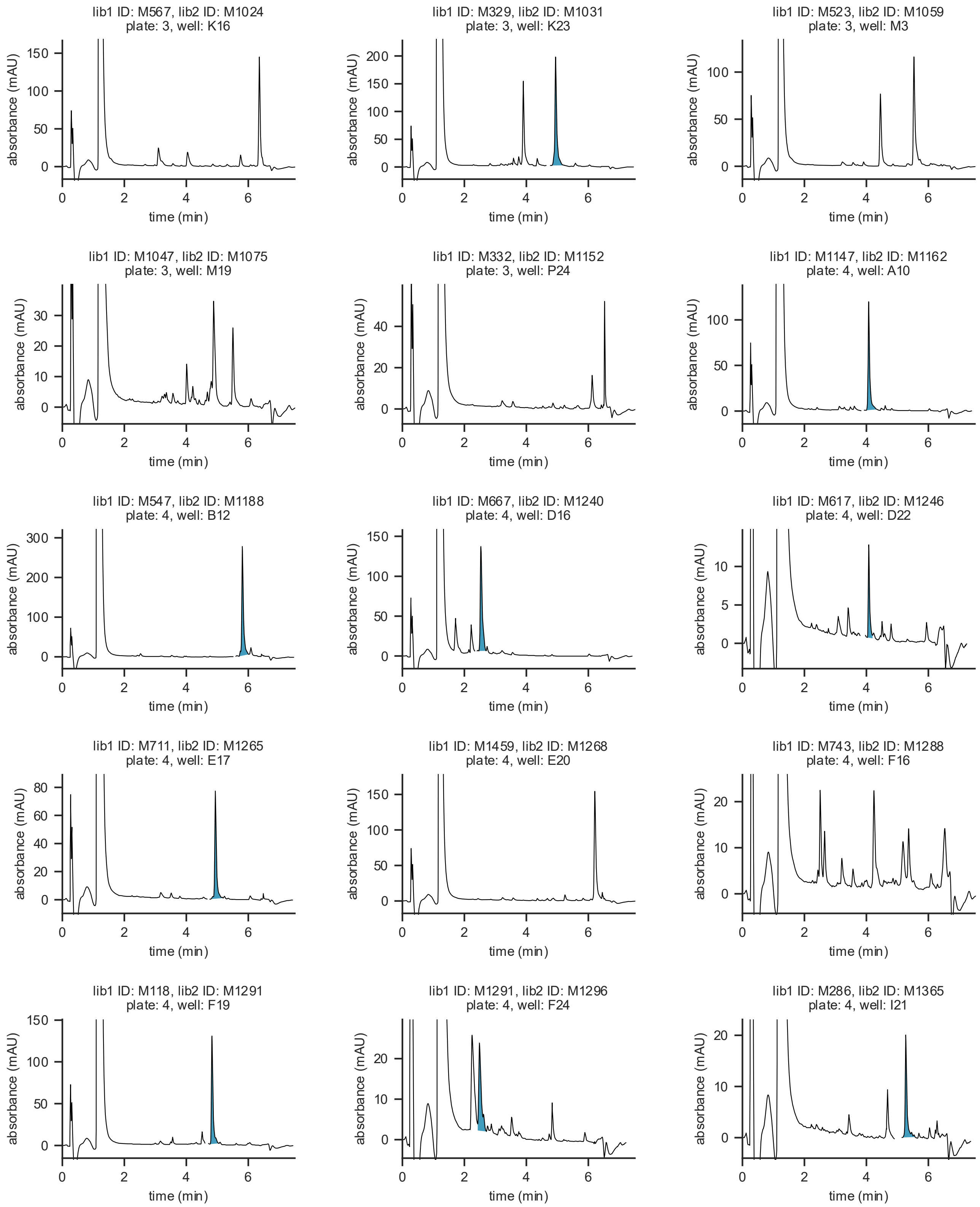

Figure S4. Continued

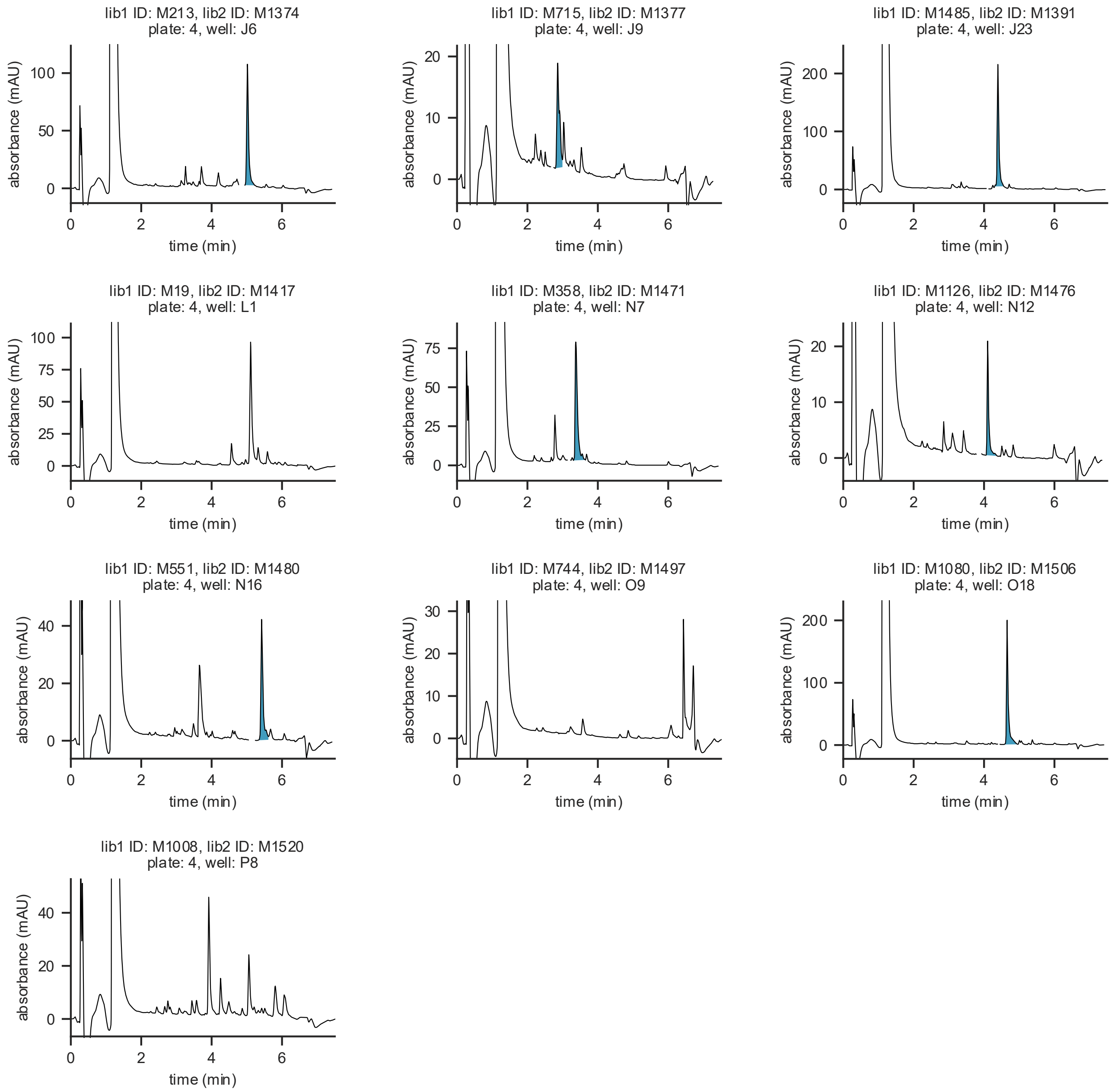

Figure S4. Continued

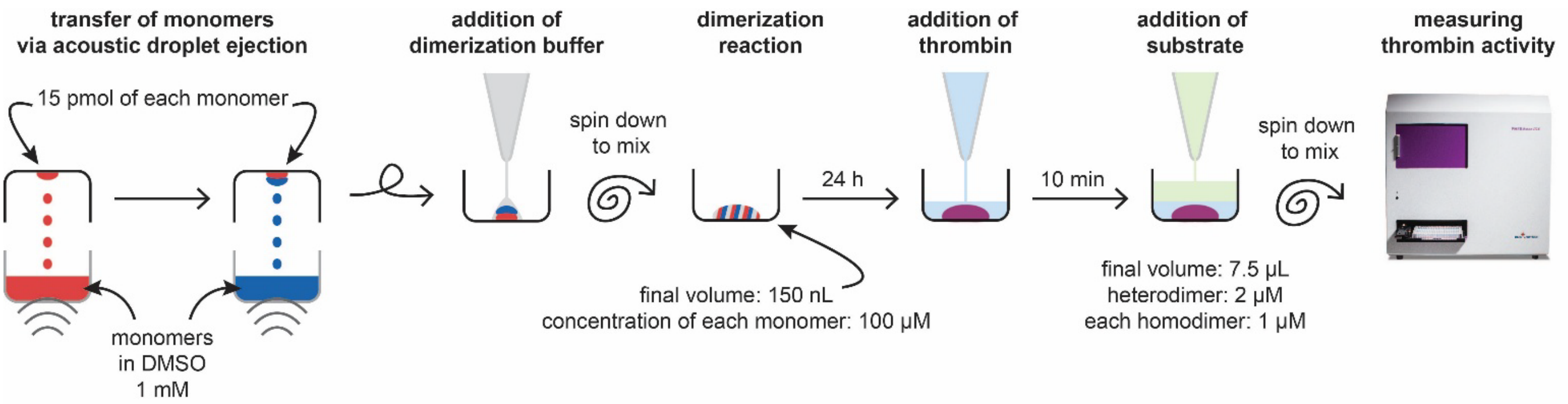

Figure S5. Schematic workflow of generation and screening of dimer Library 1.

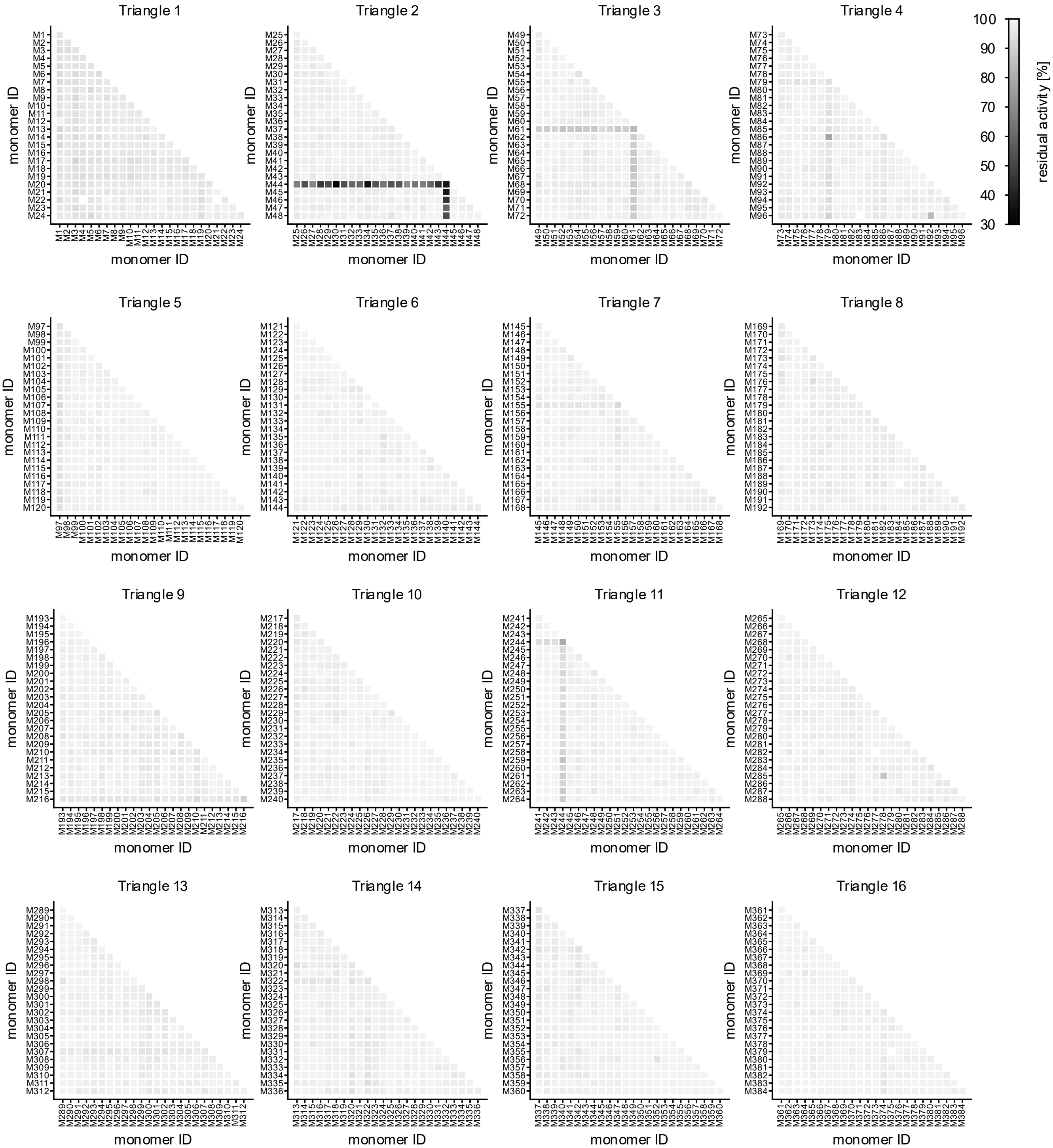

Figure S6. Screening of peptide pair Library 1 against thrombin. Volumes of 15 nL per monomer were combined via acoustic droplet ejection and incubated for 24 h at rt in 60 mM NH_4HCO_3 buffer containing 50% DMSO, with a final monomer concentration of 0.1 mM each. Assay solution was added directly to the crude product. In case of quantitative dimerization, the concentration of heterodimer was expected to be 1 µM. All data is provided as an interactive heatmap online: https://library1-interactive-heatmap.streamlit.app/.

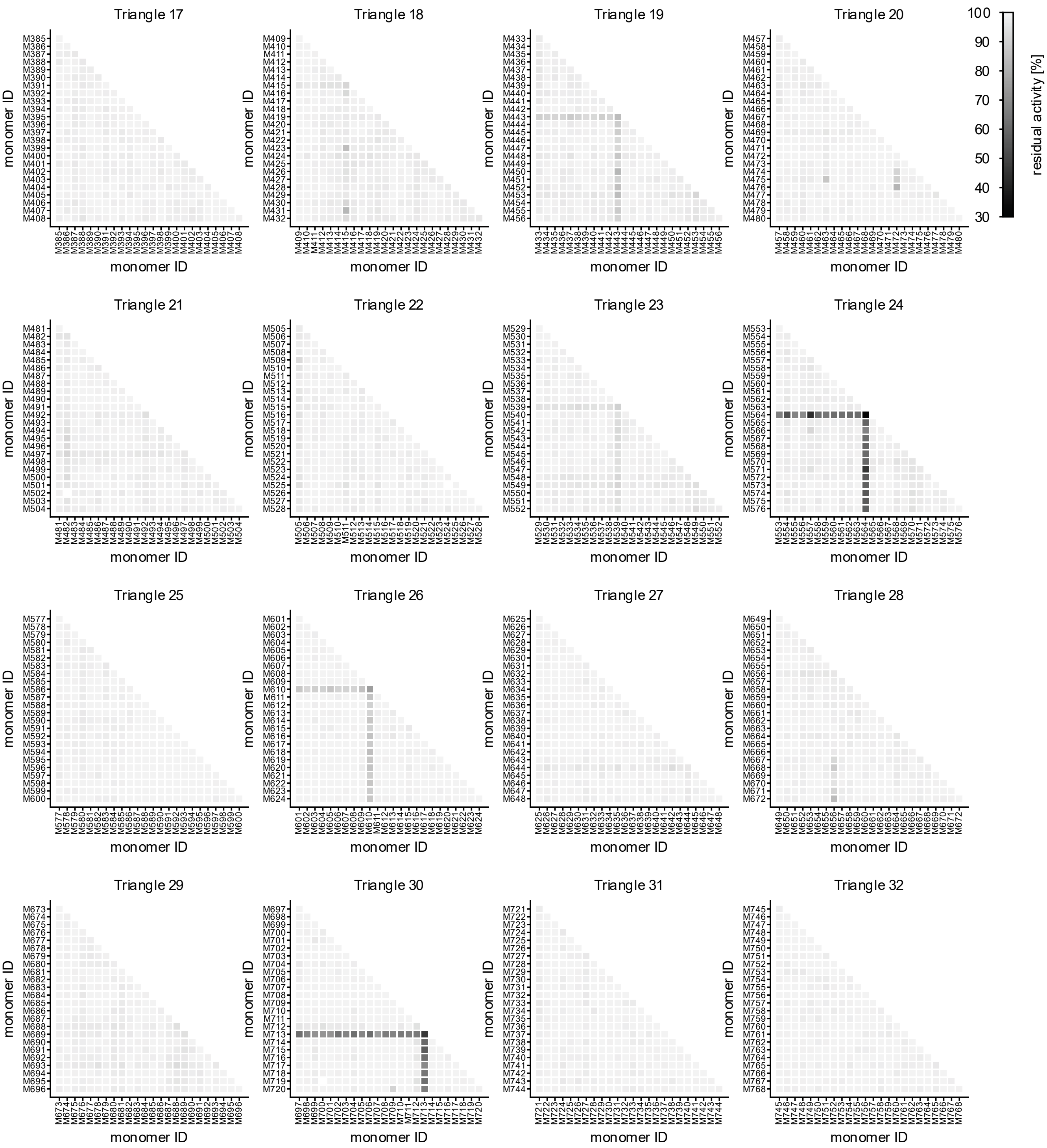

Figure S6. Continued

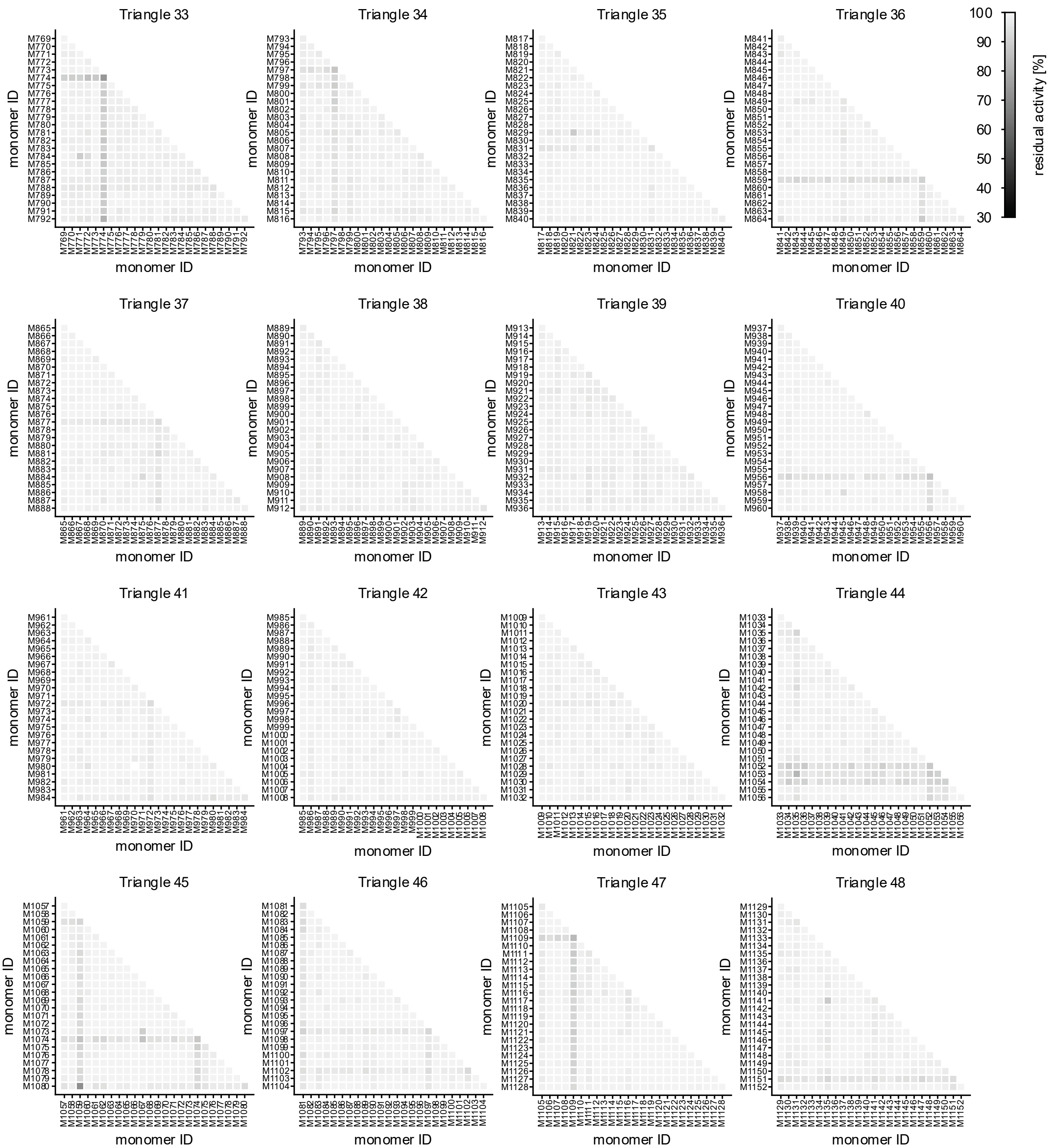

Figure S6. Continued

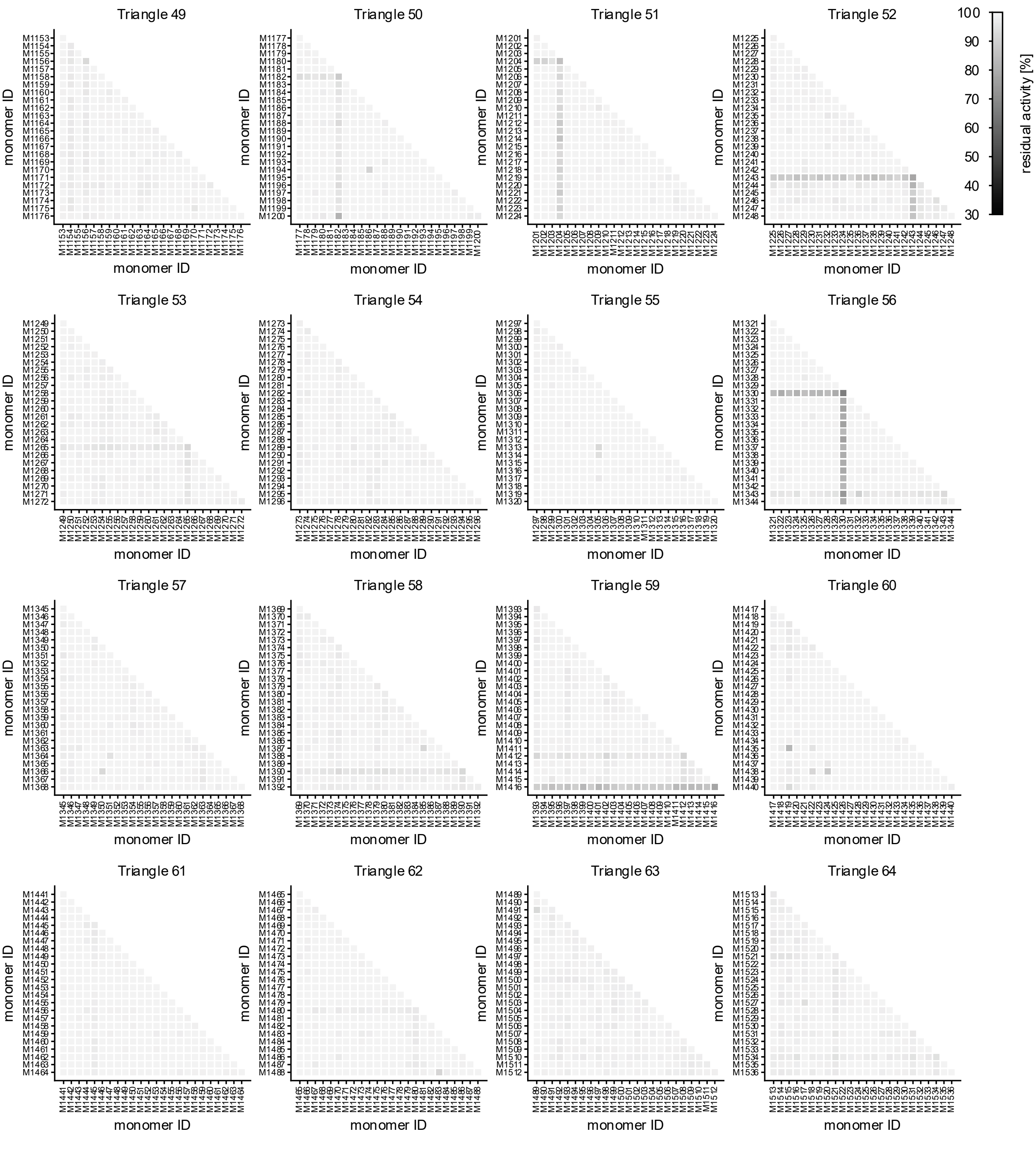

Figure S6. Continued

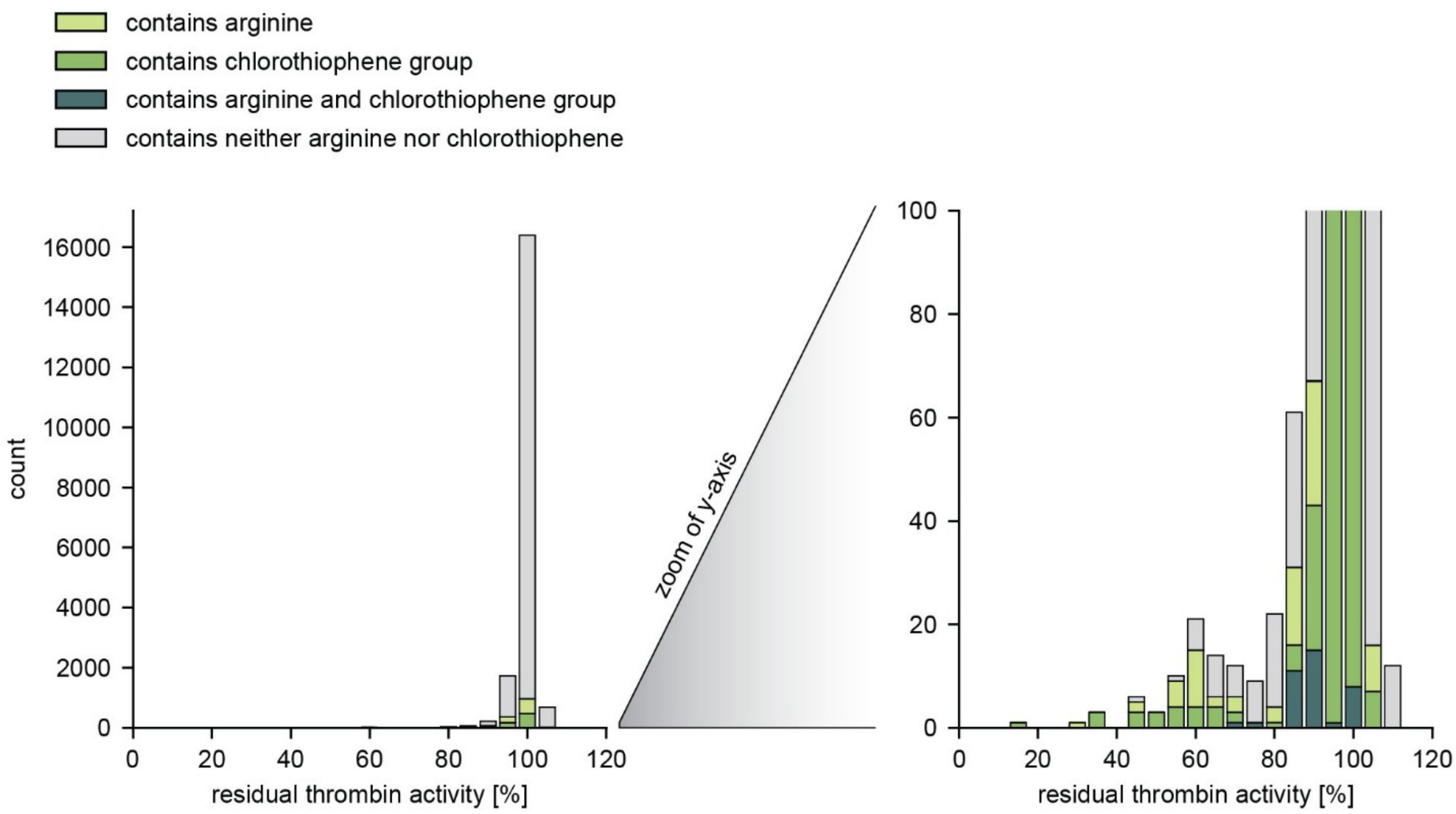

Figure S7. Abundance of guanidine and chlorothiophene groups in active peptide pairs identified in the Library 1 screen. Peptide dimers for Library 1 were grouped by occurrence of guanidinium and chlorothiophene moieties. Shown is the number of peptides pairs found for different extents of thrombin inhibition.

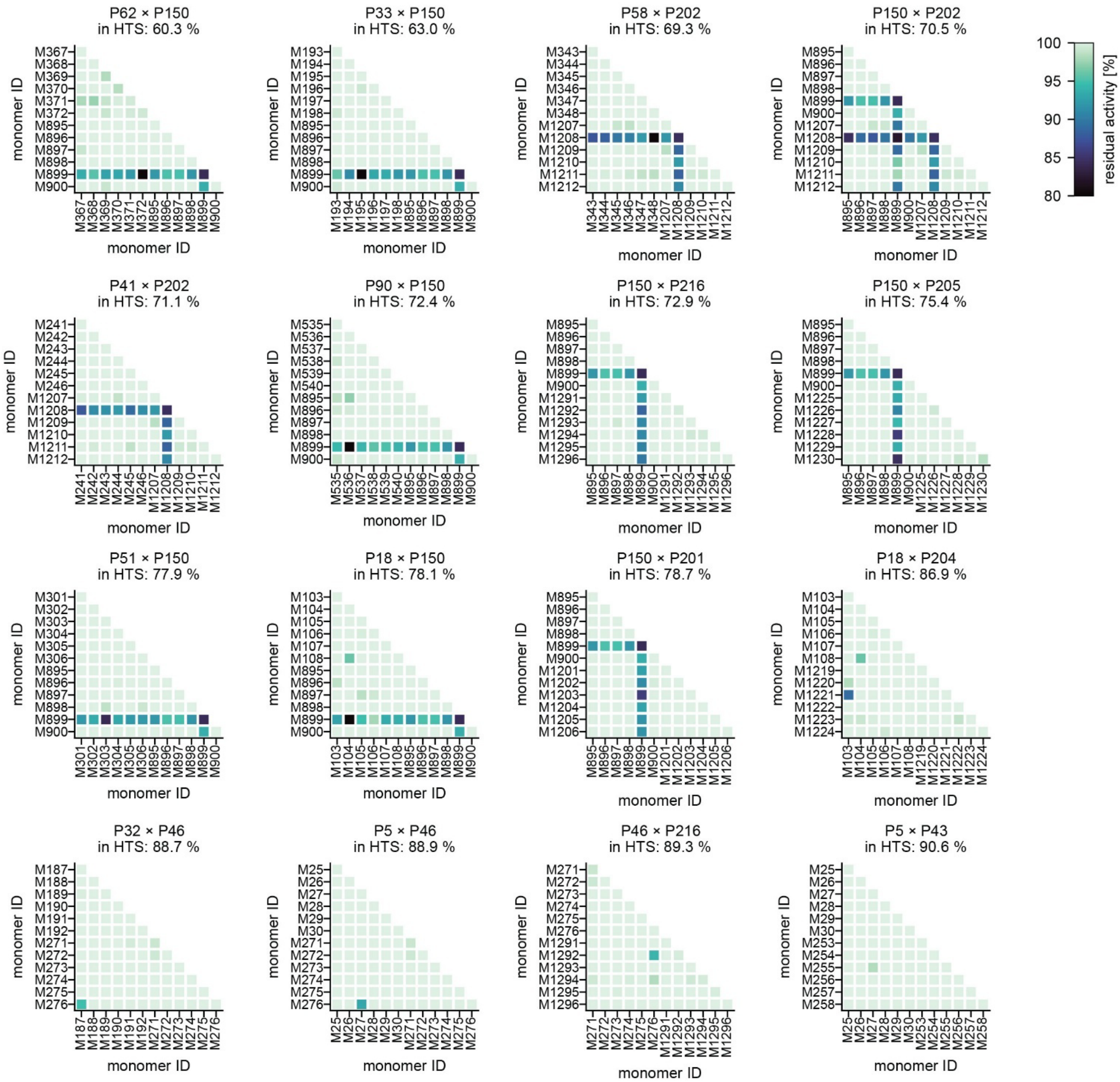

Figure S8. Deconvolution libraries of hit mixtures from the Library 2 screen against thrombin. Quantities of 2.5 pmol per monomer were combined by acoustic droplet ejection and incubated for peptide pair formation in 60 mM NH_4HCO_3 buffer containing 50% DMSO (24 h, rt). Thrombin and activity assay reagents were added directly to the reaction mixtures. Assuming that half of the monomers formed heterodimes and the other half homodimers, the concentrations of heterodimer in the thrombin inhibition assay were expected to be 167 nM.

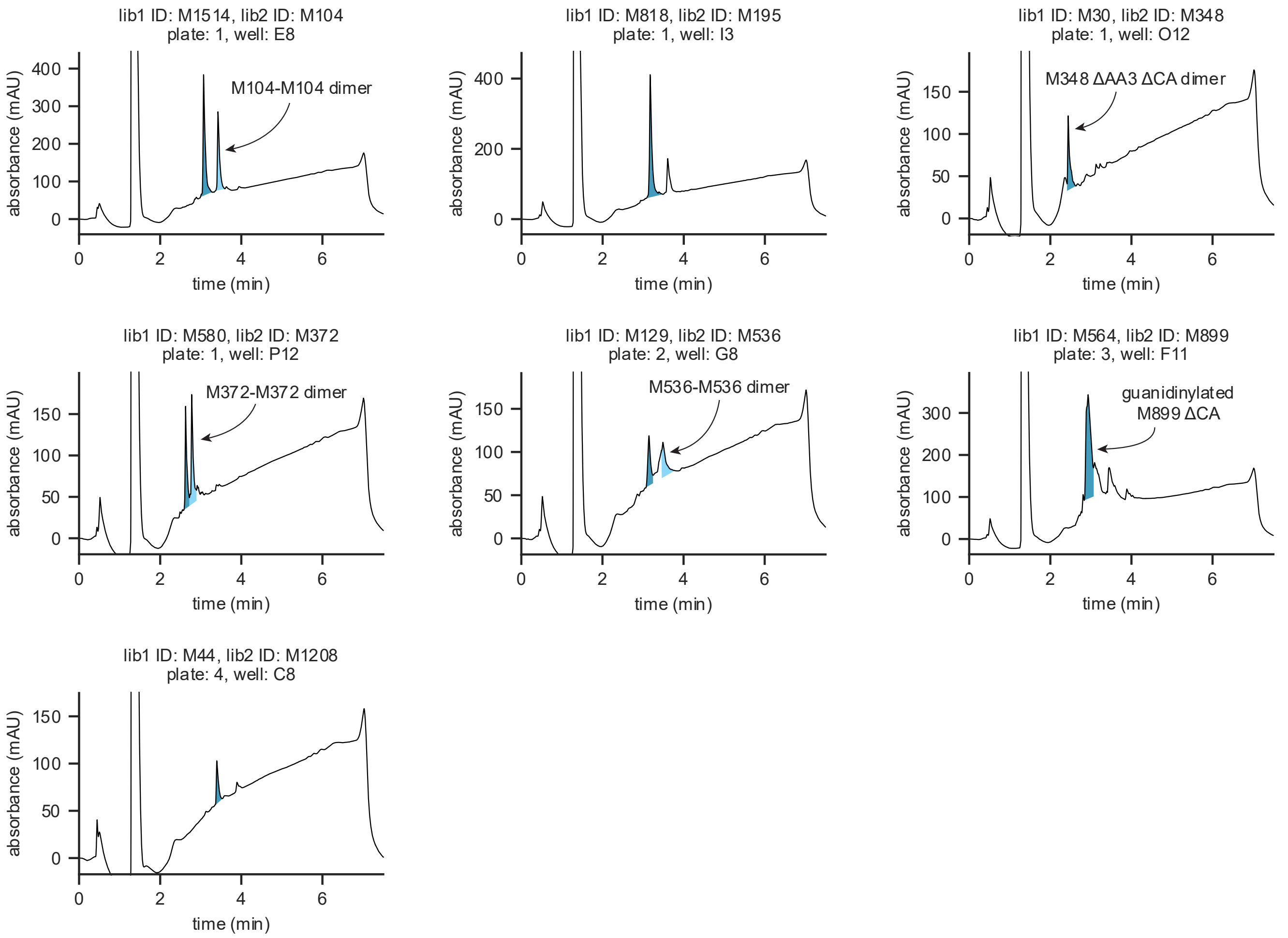

Figure S9. LC-MS analysis of thiol-peptide monomers corresponding to hits that were found in the deconvolution screen. Peptides were analyzed using a gradient of 0–100% ACN over 5 min. Gradient start time at 1 min, gradient end time at 6 min, MS detection between 1.5–6.1 min. Identified monomer is highlighted in dark blue, corresponding dimer is highlighted in light blue.

Monomers

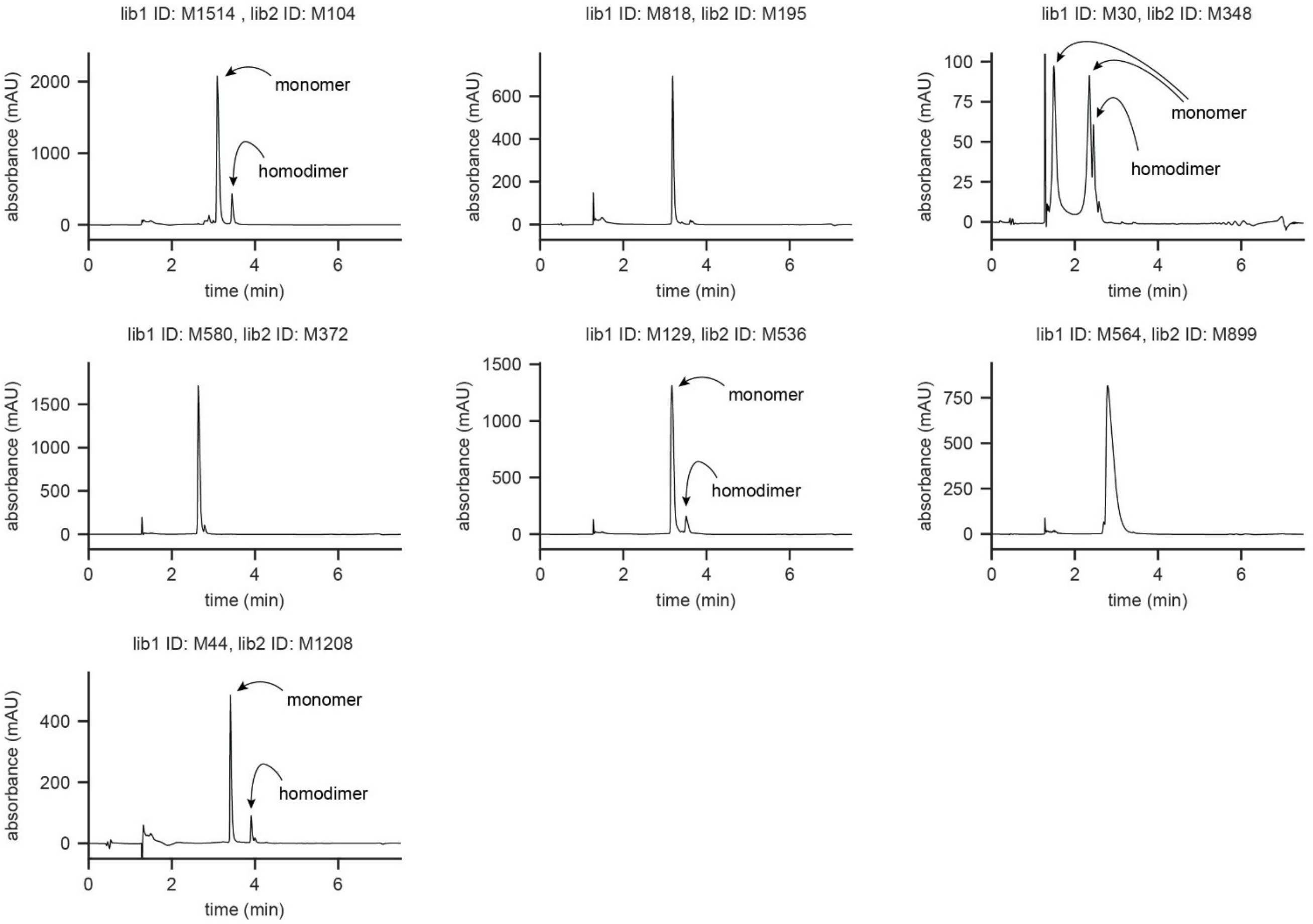

Homodimers

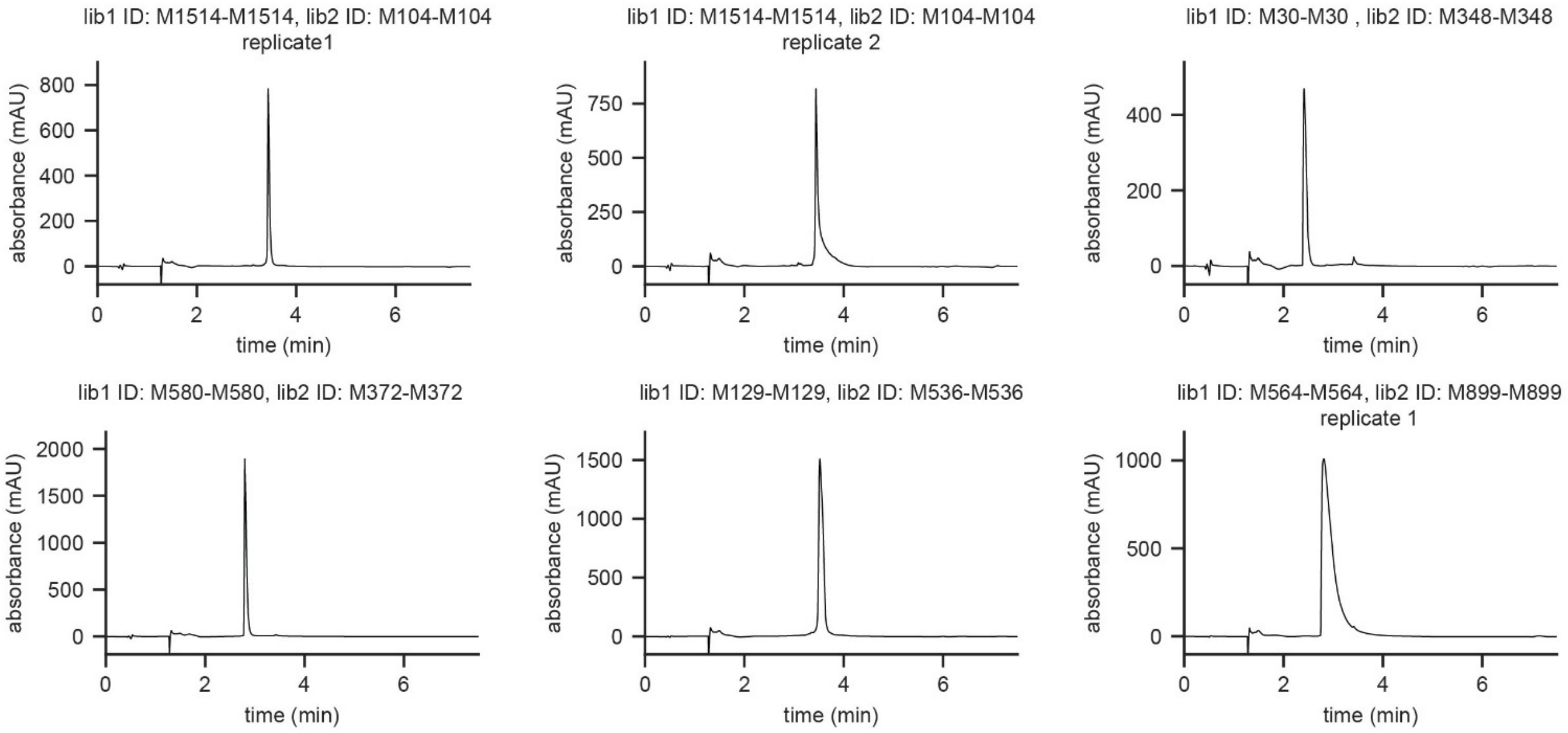

Figure S10. LC-MS chromatograms of purified compounds used for K_i determination. Compounds were analyzed using a gradient of 0–100% ACN over 5 min. Gradient start time at 1 min, gradient end time at 6 min, MS detection between 1.5–6.1 min. Some monomers stored in DMSO tended to form dimers. The activity of monomers was measured under reducing conditions to ensure that no dimers were formed.

Homodimers continued

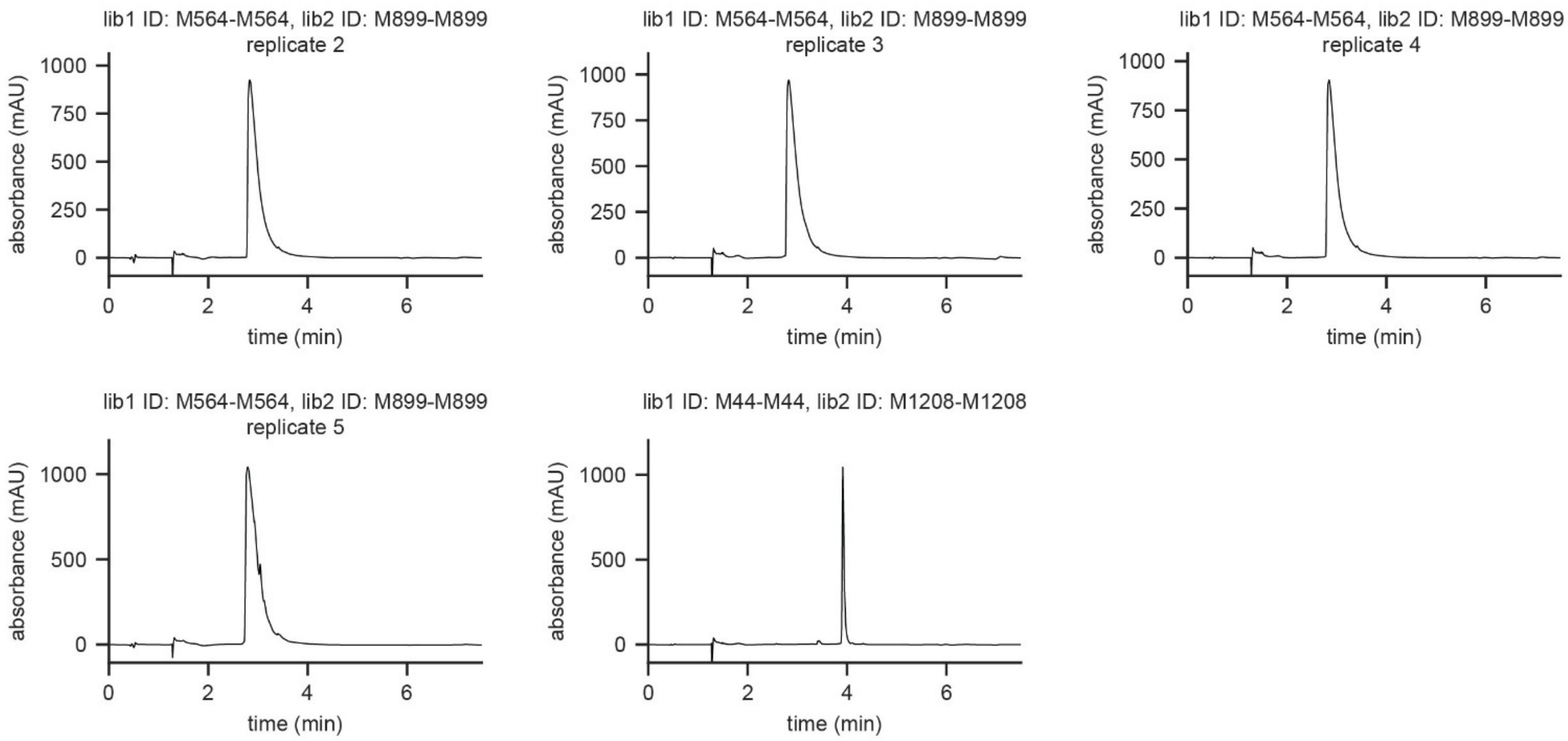

Heterodimers

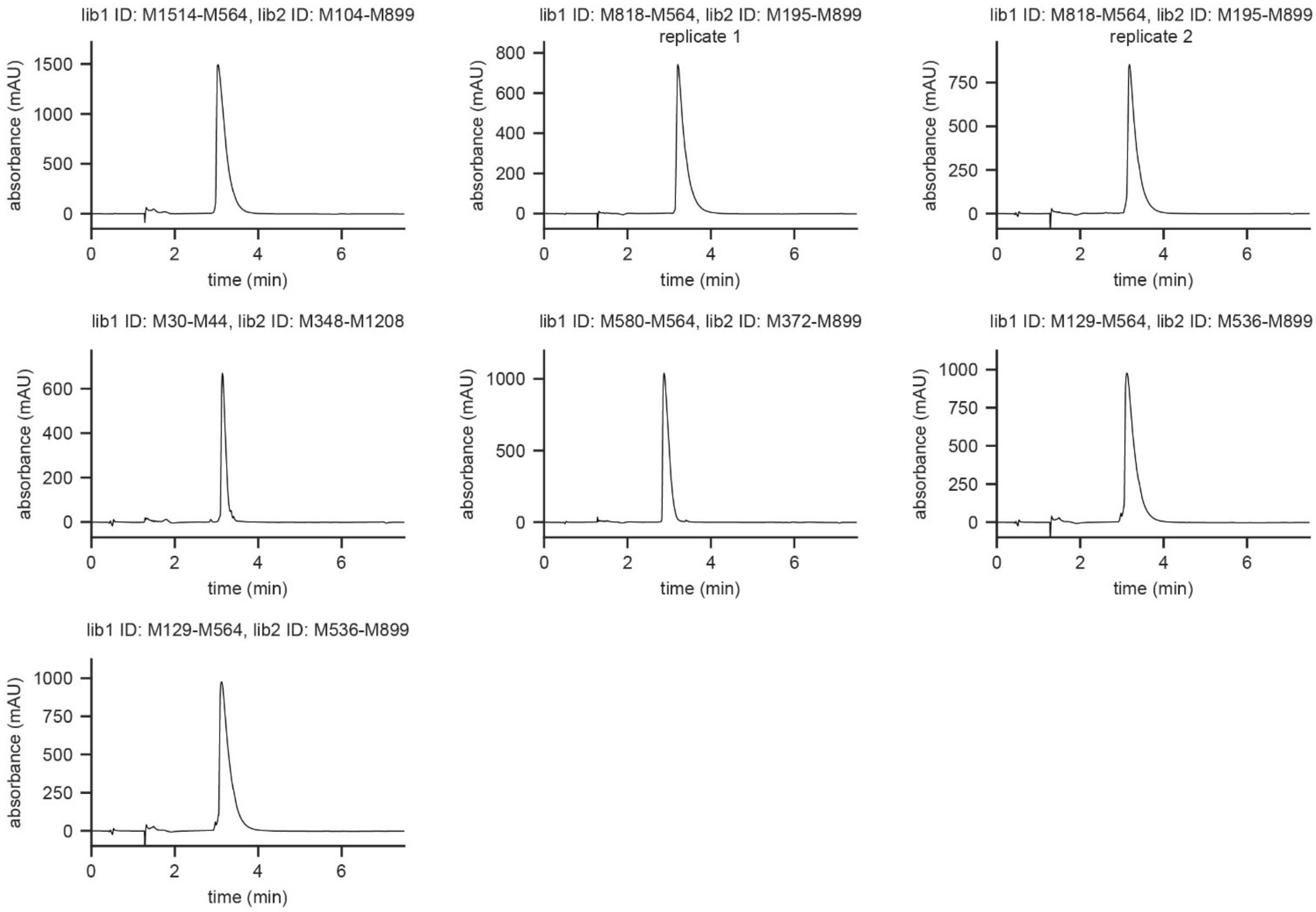

Figure S10. Continued

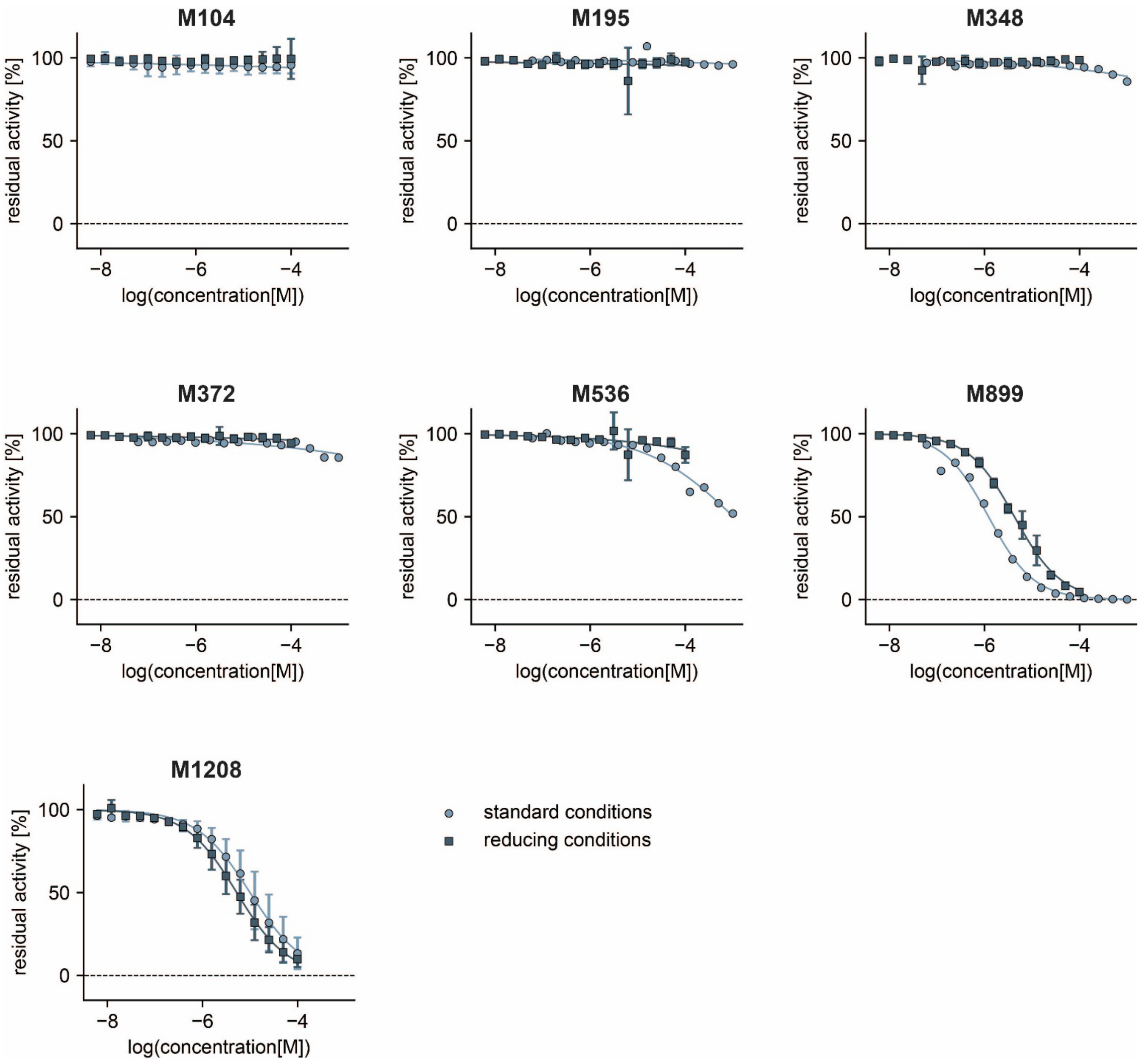

Figure S11. Dose response curves of purified monomers under standard (non-reducing) and reducing conditions. Standard conditions are indicated with circles and reducing conditions achieved with the addition of 1 mM TCEP-HCl with squares, the latter to ensure that the monomer is in its reduced form and does not oxidize to its dimer. Mean values and standard error deviations of at least three measurements (technical replicates) are indicated.

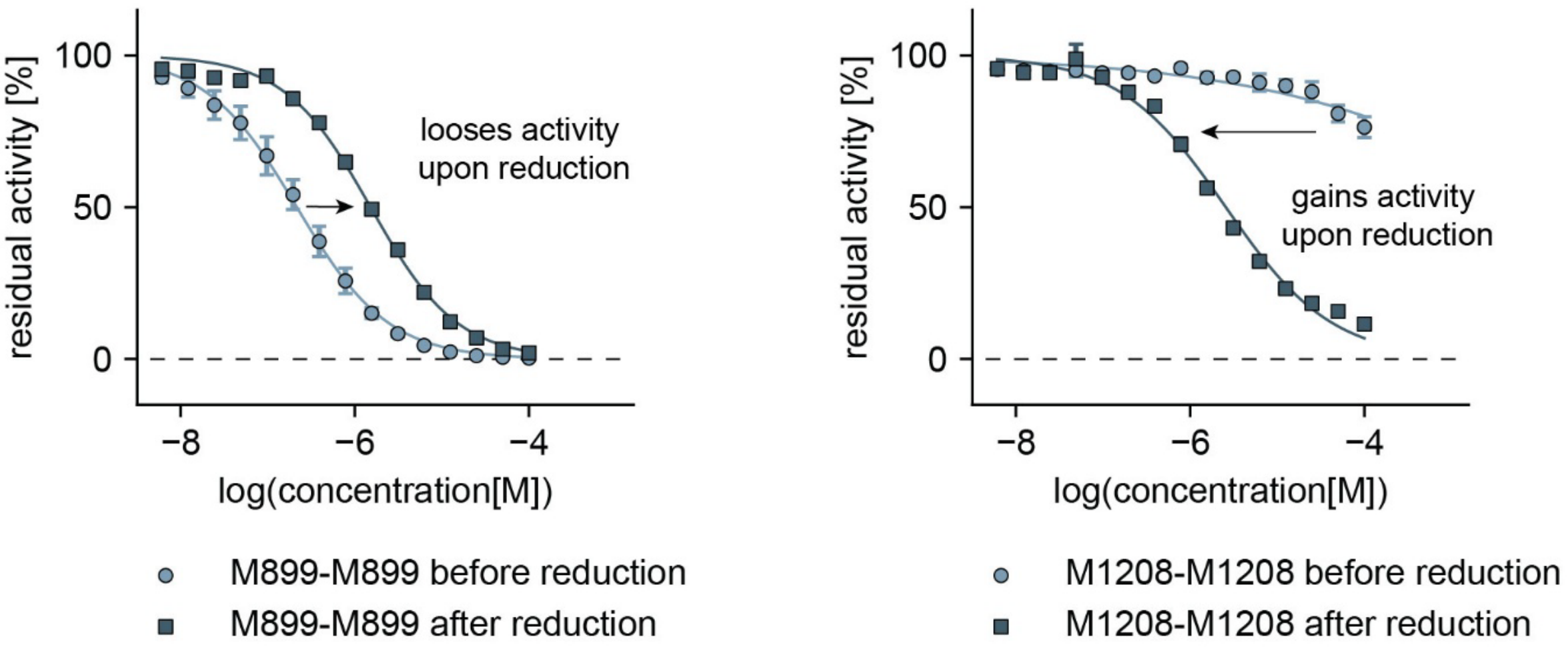

Figure S12. Change of homodimer activity upon disulfide bridge reduction. Dose response curves and K_i values of purified M899-M899 and M1208-M1208 homodimers before and after reduction. Reducing conditions were achieved with addition of 1 mM TCEP-HCl to the assay buffer. The homodimer concentrations are indicated on the x-axis. Mean values and standard error deviations of at least three measurements (technical replicates) are indicated.

ABBREVIATIONS

ACN	acetonitrile
BDT	butanedithiol
BSA	bovine serum albumin
DCM	dichloromethane
DMF	N,N-dimethylformamide
DMSO	dimethyl sulfoxide
DTNB	dithionitrobenzoic acid
Fmoc	fluorenylmethoxycarbonyl
HATU	1-[bis(dimethylamino)methylene]-1H-1,2,3-triazolo[4,5-b]pyridinium 3-oxide hexafluorophosphate
IC_{50}	half maximal inhibitor concentration
K_i	inhibitory constant
LC-MS	liquid chromatography-mass sepctrometry
NMM	N-methylmorpholine
NMP	N-methylpyrrolidone
RP-HPLC	reverse phase-high performance liquid chromatography
rt	room temperature
RVC	rotational vacuum concentration
SPPS	solid-phase peptide synthesis
TCEP-HCl	tris(2-carboxyethyl)phosphine hydrochloride
TEA	triethylamine
TFA	trifluoroacetic acid
TIPS	triisopropylsilane
